# Improved Functionality of Exhausted Intrahepatic CXCR5+ CD8+ T Cells Contributes to Chronic Antigen Clearance Upon Immunomodulation

**DOI:** 10.3389/fimmu.2020.592328

**Published:** 2021-02-03

**Authors:** Kingsley Gideon Kumashie, Marcin Cebula, Claudia Hagedorn, Florian Kreppel, Marina C. Pils, Friedrich Koch-Nolte, Björn Rissiek, Dagmar Wirth

**Affiliations:** ^1^ Model Systems for Infection and Immunity, Helmholtz Centre for Infection Research, Braunschweig, Germany; ^2^ Chair of Biochemistry and Molecular Medicine, University Witten/Herdecke, Witten, Germany; ^3^ Mouse Pathology Unit, Helmholtz Centre for Infection Research, Braunschweig, Germany; ^4^ Institute of Immunology, University Medical Center Hamburg-Eppendorf, Hamburg, Germany; ^5^ Institute of Neurology, University Medical Center Hamburg-Eppendorf, Hamburg, Germany; ^6^ Institute of Experimental Hematology, Hannover Medical School, Hannover, Germany

**Keywords:** T cell exhaustion, CXCR5+ T cells, CpG oligonucleotide, T cell reinvigoration, exhausted stem-like T cells, liver resident T cells, follicular helper-like T cells, liver

## Abstract

Chronic hepatotropic viral infections are characterized by exhausted CD8+ T cells in the presence of cognate antigen in the liver. The impairment of T cell response limits the control of chronic hepatotropic viruses. Immune-modulatory strategies are attractive options to re-invigorate exhausted T cells. However, in hepatotropic viral infections, the knowledge about immune-modulatory effects on the in-situ regulation of exhausted intrahepatic CD8+ T cells is limited. In this study, we elucidated the functional heterogeneity in the pool of exhausted CD8+ T cells in the liver of mice expressing the model antigen Ova in a fraction of hepatocytes. We found a subpopulation of intrahepatic CXCR5+ Ova-specific CD8+ T cells, which are profoundly cytotoxic, exhibiting efficient metabolic functions as well as improved memory recall and self-maintenance. The intrahepatic Ova-specific CXCR5+ CD8+ T cells are possibly tissue resident cells, which may rely largely on OXPHOS and glycolysis to fuel their cellular processes. Importantly, host conditioning with CpG oligonucleotide reinvigorates and promotes exhausted T cell expansion, facilitating complete antigen eradication. The CpG oligonucleotide-mediated reinvigoration may support resident memory T cell formation and the maintenance of CXCR5+ Ova-specific CD8+ T cells in the liver. These findings suggest that CpG oligodinucleotide may preferentially target CXCR5+ CD8+ T cells for expansion to facilitate the revival of exhausted T cells. Thus, therapeutic strategies aiming to expand CXCR5+ CD8+ T cells might provide a novel approach against chronic liver infection.

## Introduction

CD8+ T cells are central to the control of viral infection by producing effector molecules such as granzyme B (GZMB), interferon gamma (IFN-γ) and tumor necrosis factor (TNF-α) (1–3). During acute infection, CD8+ T cells kill antigen expressing cells, facilitating long-lived memory T cell formation (1–3). Contrarily, in chronic liver infections such as hepatitis B virus (HBV) and hepatitis C virus (HCV) infections, memory T cells fail to develop, due to T cell exhaustion (4, 5). Exhausted T cells are marked by poor effector function and co-expression of multiple inhibitory receptors such as PD-1 and TIM-3, as well as deranged epigenetic, metabolic and transcriptional signatures (6–8). The persistence and duration of antigen exposure are important key features known to drive the development of exhausted T cells (8). Accordingly, immunomodulatory strategies that revive exhausted T cells *via* boosting the functionality and formation of tissue resident T cells are considered as an attractive therapeutic option to combat chronic liver infection. Indeed, T cell inhibitory receptor PD-1 blocking strategies have emerged that improves exhausted T cell function (8). Also, strategies triggering Toll-like receptor 9 (TLR9) signaling on myeloid cells have been proposed recently to improve exhausted T cell function (9). TLR9 ligand CpG ODN, which induces inflammation and promotes the maturation of myeloid cells has been suggested to boost T cell function in the presence of persistent liver antigen (9).

In models of chronic LCMV infection, exhausted T cells have been shown to be functionally and phenotypically heterogeneous (3). These heterogeneous lineages are made of progenitor CXCR5+ and terminal CXCR5- CD8+ T cell subsets (10–13). The CXCR5+ CD8+ T cells are stem-like cells which generate terminally exhausted CXCR5- CD8+ T cells. In addition, the CXCR5+ CD8+ T cells are highly responsive to PD-1 blockade (10, 11). In contrast, the CXCR5-CD8+ T cells are less responsive to PD-1 therapy and lack the ability to differentiate into CXCR5+ CD8+ T cells. On a molecular level, TCF-1 is critical for long-term maintenance of the CXCR5+ CD8+ T cells (10, 14, 15). Strikingly, different tissue localization has been described for CXCR5+ and CXCR5- CD8+ T cells. The terminal CXCR5- CD8+ T cells were profound in peripheral tissues and lymphoid organs while CXCR5+ CD8+ T cells are preferentially in lymphoid organs – the site of LCMV infection (10, 11). The CXCR5 ligand, CXCL13, is predominantly expressed from cells in the lymphoid organ (16). While the initial insight into exhausted T cells was obtained from chronic LCMV infections, similar phenomena have been reported in patients with chronic HBV and HCV infections. However, most studies evaluating exhausted T cell responses in chronic HBV and HCV patients rely on peripheral T cells profiling, despite viral replication in the liver (17). Thus, these studies do not definitively demonstrate the phenotype of exhausted T cell at the site of antigen exposure. Moreover, the dynamic role CXCR5+ CD8+ T cells play in chronic liver infection is yet to be fully explored.

A deeper understanding of exhausted T cells in chronic liver infection is hindered by the lack of an appropriate infection model that recapitulates HBV and HCV infection. While overexpression of human specific entry factors (18) and elimination of mouse restriction factors (19) recently facilitated HCV infection in mice, it remains to be elucidated if these models can also reflect late infection states, i.e., the establishment of chronic infections and corresponding/accompanying diseases such as liver fibrosis, cirrhosis and eventually liver failure.

The liver has a unique microenvironment and can function as a lymphoid organ, which is distinct from classical lymphoid tissues. Among various immunomodulatory functions, the liver has the propensity to prime functional CD8+ T cell immunity (20–22). We previously introduced a transgenic mouse model in which intracellular Ovalbumin (Ova) expression is activated by Tamoxifen (Tam) inducible CreERT2 recombinase (23, 24), which allows for elucidating T cell responses towards Ova antigen in defined tissues (25–28). Using OvaXCre mice, in which CreERT2 is controlled by the albumin promoter, we documented that the frequency of Ova expressing hepatocytes can be adjusted by Tam titration (23, 25–27). Moreover, we demonstrated that adoptive transfer of Ova-specific CD8+ T cells (OT-1 cells) or Ova-specific CD8+ T cell induction by vaccination eliminates low frequencies of Ova expressing hepatocytes. In contrast, when Ova-specific CD8+ T cells are confronted with elevated frequencies of Ova expressing hepatocytes (high antigen conditions), CD8+ T cells lose their cytotoxic activity and rather exhibit an exhausted phenotype, characterized by the expression of multiple exhaustion markers such as Tim-3, PD-1 and Lag-3 (29, 30). Thus, the high antigen condition in the OvaXCre model mimics the situation in early stage chronic hepatotropic viral infection, characterized by persistent viral antigen expression that drives exhausted T cell development, in the absence of end-stage liver diseases such as fibrosis, cirrhosis and finally cancer.

Here, using the OvaXCre mouse model, we show that CXCR5+ CD8+ T cells are also generated upon Ova antigen recognition in the liver. Importantly, the intrahepatic Ova-specific CXCR5+ CD8+ T cells preferentially express TCF-1 and Bcl6. The CXCR5+ CD8+ T cells are endowed with potent cytotoxic functions, an extensive proliferation rate as well as efficient mitochondria and nutrient uptake function. In addition, the CXCR5+ Ova-specific CD8+ T cells show profound *in vivo* and *in vitro* maintenance, survival, recall responses and self-renewal. Importantly, we show that CpG ODN host conditioning reinvigorates exhausted Ova-specific T cells and promotes the formation of T cells that show markers of liver residency. Accordingly, CpG ODN potentiates increased CXCR5+, TCF-1+ and CD62L+ T cell numbers in the liver. Finally, CpG ODN treatment facilitates the enrichment of GZMB in CXCR5+ and CXCR5- Ova-specific CD8+ T cells, which fosters antigen clearance. In conclusion, CXCR5+ Ova-specific CD8+ T cells may be the subset of cells preferentially targeted by CpG ODN treatment to mediate reinvigoration of exhausted T cells.

## Experimental Procedures

### Mice and Cell Isolation

The design of the transgenic OvaXCre mice deployed for this study is depicted in **Figure S1** and was previously described (23, 24, 29). The OvaXCre mice were administered with 50 µg Tamoxifen (Tam; Ratiopharm, Ulm, Germany) to induce high Ova antigen, as described elsewhere (24). Tam treated mice were used two weeks post treatment for all experiments. All experiments were performed with mice 6–20 weeks of age. The mice were bred in-house in individually ventilated cages and maintained under specific pathogen-free conditions. OT-1 cells used for adoptive transfer were isolated from the spleen of Thy1.1 OT-1 or dsRED OT-1 mice, as previously described (24). These OT-1 mice were generated on a C57Bl/6 background. Isolation of single cell suspension of liver non-parenchyma cells were performed as described elsewhere (24) but without *in situ* liver digestion step.

### Adoptive OT-1 Transfer and Vaccination of Mice

For adoptive transfer Thy1.1+ OT-1 and dsRED+ OT-1 cells were purified using negative CD8a+ T cells isolation kit (Miltenyi Biotec, Germany), according to the manufacturer’s protocol. The adoptive transfer procedure was described previously (24) and in all experiments, 3–5 x 10^6^ cells were transferred per high antigen mouse. Vaccination was performed with an adenoviral vector encoding OVA antigen (AdOva). The AdOva vector (30–32) and the vaccination protocol were previously described (30). In addition, administration of CpG ODN was performed as described elsewhere (30). Briefly, CpG ODN was reconstituted to 0.2μg/μl in 0.9 NaCl and 100μl was *i.v* injected into recipient mice on days 13, 16, 20, 24, and 28 post OT-1 transfer.

### Serum Isolation for CXCL13 ELISA Assay

Serum was isolated from 100 µl of blood collected from each mouse. CXCL13 was quantified using the murine BLC (CXCL13) ELISA kit (Thermo Scientific) according to the manufacturer’s protocol. The absorbance was measured with TriStar ELISA plate reader (Berthold Technologies, Germany) at 450 nm. The concentration of serum CXCL13 was interpolated from the standard curve plotted.

### RNA Isolation and qRT-PCR

RNA isolation was performed as previously described (24) and 2 µg of total RNA were reverse transcribed using the RevertAid First strand cDNA synthesis kit (ThermoFischer Scientific), according to the manufacturer’s protocol. The qRT-PCR for Ova was performed using primer pairs outlined elsewhere (24) and normalized to the expression of albumin.

### Flow Cytometry

OT-1 cells were identified based on Thy1.1+ or dsRED expression. Vaccination induced Ova specific T cells were identified by Ova pentamer staining according to a previously published protocol (30). To stain for extracellular markers, Fc receptors on single cell suspension of liver non-parenchyma cells were blocked with anti-mouse CD16/32 (produced in-house) and stained with indicated antibodies (**Supplementary Table**) for 1 h at 4°C. Dead cells were excluded with Live/Dead (1/1,000 in PBS, ThermoFischer Scientific) according to the manufacturer’s protocol. Prior to staining for transcription factors (**Supplementary Table**), cells were fixed and permeabilized with FoxP3/transcription factor staining kit (eBiosciences), according to the manufacturer’s protocol. For intracellular staining (**Supplementary Table**), single cells were re-stimulated 5 h *ex vivo* in RPMI complete media (RPMI media, 5% FCS, 1% Pen/Strep, 1% Glutamine, 1mM HEPES) supplemented with PMA/Ionomycin (10 ng/ml PMA and 1µg/ml Ionomycin) with Brefeldin A for the last 2 h of re-stimulation. Prior to intracellular staining, the stimulated cells were extracellularly stained with antibodies, fixed and permeabilized using Cytofix/Cytoperm kit (BD Biosciences) according to the manufacturer’s protocol. For CD107a staining, CD107a was reconstituted with liver non-parenchyma cells and *ex vivo* re-stimulated with PMA/Ionomycin as previously described. Data were collected on LSRII or LSR Fortessa (BD Biosciences) and analyzed using FlowJo software (v10.6.0; BD Pharmingen). In this study, all flow cytometry gates were defined for the expression of positive markers of interest using fluorescence minus one (FMO) controls on the same cell batch. The gating strategy employed to identify Ova-antigen specific CD8+ T cells is shown in **Figure S1C, D**.

### Glucose Uptake

50–100 µM of 2-Deoxy-2-[(7-nitro-2,1,3-benzoxadiazol-4-yl) amino]-D-glucose (2-NBDG, Abcam) was reconstituted in RPMI media (without nutrient supplement). 100 µl of the reconstituted 2-NBDG was aliquoted onto 5 x 10^5^ liver non-parenchyma cells in a 96 well flat bottom plate and cells were cultured for 45–60 min (5% CO_2_, 37°C). Cells were washed and stained for antigen specific CD8+ CXCR5+ T cells for flow cytometry. Data were collected and analyzed by flow cytometry as previously described.

### Mitochondria Staining

Specific mitochondria sensitive dyes were reconstituted in RPMI media as follows: 50 nM Mitotracker Green (MTG; Invitrogen), 25 nM Mitotracker Deep Red (MTDR; Invitrogen), 300 nM TMRE and 5 µM 2′,7′-Dichlorofluorescin diacetate (DCFDA; Invitrogen). 100 µl each of the reconstituted dye was incubated with 5 x 10^5^ liver non-parenchyma cells in a 96 well flat-bottom plate for 30 min (37°C, 5% CO_2_). After the incubation, cells were washed and stained for antigen specific CD8+ CXCR5+ T cells for flow cytometry. Data were collected and analyzed by flow cytometry as previously described.

### Sorting and Adoptive Transfer of CXCR5+ and CXCR5- CD8+ T Cells

For the purpose of isolating CXCR5+ and CXCR5- CD8+ T cells, dsRED OT-1 cells were transferred into high antigen mice. D9 post OT-1 transfer, 150 µl of 0.133 µg/µl (in 0.9% NaCl) ARTC2.2 nanobody was injected *i.v*. Liver non-parenchyma cells were isolated 30 min later, reconstituted with Fc receptor blocking antibody and stained for CXCR5, B220, CD8 antibodies as well as Live/Dead dye. Sorting was performed by gating on Live dsRED+CD8+B220- OT-1 cells. The dsRED+OT-1 cells were further sorted into CXCR5+ and CXCR5- CD8+ T cell subsets using LSR Fusion and LSR Aria (BD Biosciences). For adoptive transfer, 1 x 10^5^ CXCR5+ or CXCR5- CD8+ T cells in 200 µl of PBS were transferred *i.v.* into low antigen recipient mice.

### *In Vitro* Proliferation, Conversion, and Cytotoxicity Assay

To compare the cytotoxic function of CXCR5+ and CXCR5- CD8+ T cells, 6 x 10^5^ murine EL4 tumor cells were labelled with 2.5 µM (high) or 0.25 µM (low) CFSE (CFSE^hi^ and CFSE^lo^). The EL4 CFSE^hi^ cells, which served as the target cells, were pulsed with 2 µg/ml peptide for 1.5 h in a 37°C water bath. The CFSE^lo^ cells were incubated for the same time without SIINFEKL peptide. 4,000 EL4 cells each of CFSE^hi^ and CFSE^lo^ were plated in a 96 well U-bottom plate and co-cultured with CXCR5+ or CXCR5- CD8+ T cells isolated from high antigen mice on D14 post OT-1 transfer. The co-cultured cells were incubated at 37°C for 24 h using an effector:target ratios of 2:1 and 5:1. The cells were washed and analyzed by flow cytometry. The rate of CFSE^hi^ EL4 cell killing was normalized to the CFSE^lo^ EL4 cells in each well.

*In vitro* proliferation and conversion assays were performed on CXCR5+ and CXCR5- CD8+ T cells isolated on D9 post OT-1 transfer into high antigen mice. The sorted cells were labelled with 5µM CFSE and stimulated *in vitro* with Dynabead anti-CD3/28 (ThermoFischer Scientific), supplemented with 100 U/ml IL-2 (PeproTech, Germany), based on a protocol previously published for progenitor and terminally exhausted T cells in tumor (33). Similar labelling of CXCR5+ and CXCR5- CD8+ T cells with CFSE was confirmed with naïve OT-1 cells. Approximately, 6 x 10^4^ CXCR5+ or CXCR5- CD8+ T cells were stimulated for 48 h at 37°C/5% CO_2_. The cells were washed, stained and analyzed by flow cytometry as previously described.

### Statistical Analysis

All graphs were plotted with, and statistical differences were calculated in GraphPad using Mann-Whitney’s test. Each data point (dot) on the graph represents a biological replicate and the error bars were computed as: Mean ± SEM. The p values ≤0.05 (*), ≤0.01 (**), or ≤0.001 (***) are representative of significant differences between two groups and non-significant (p >0.05) differences were indicated as ‘ns’.

## Results

### Exhausted Intrahepatic Ova-Specific CD8+ T Cells Harbor CXCR5+ CD8+ T Cells

To investigate the dynamic regulation of exhausted CD8+ T cell subsets in the liver, we employed the OvaXCre mouse model upon induction with Tam, a condition in which about 50% of hepatocytes express the intracellular, non-secreted model antigen Ova (24) (see **Figure S1A** for the description of the model). In this condition (so-called high antigen) adoptive transfer of antigen specific T cells or vaccination results in functionally impaired T cells (29, 30). Upon vaccination with an adenoviral vector encoding ovalbumin (AdOva) or upon adoptive transfer of antigen specific CD8+ T cells, we observed accumulation of Ova-antigen specific T cells in the liver (**Figure S1B, D**). The frequency of Ova- specific CD8+ T cells in the liver increased over time and plateaued to ca. 8%–10% from D14 (**Figure S1D**) while the absolute numbers increased until D9 post vaccination and decreased thereafter (**Figure S1E**). These Ova- specific CD8+ T cells were previously shown to be functionally impaired, characterized by severe exhaustion (29, 30). Of note, the antigen specific CD8+ T cells in the liver failed to eradicate the Ova antigen load (**Figure S1F**). These data demonstrate that the presence of persistent antigen in the liver induces functional impairment and subsequent depletion of Ova-specific CD8+ T cells.

**Figure 1:**
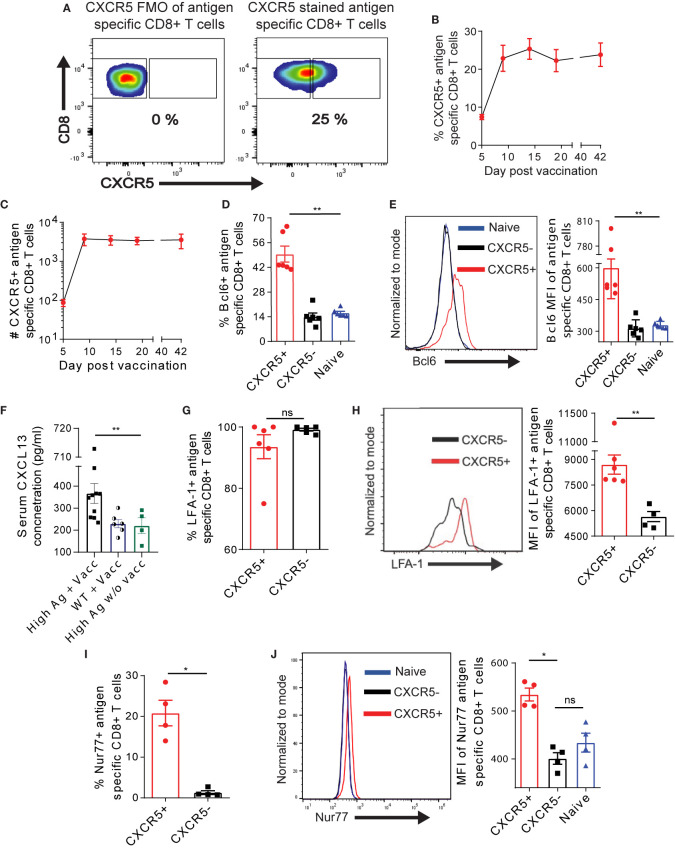
Exhausted Ova-specific CD8+ T cells in the liver harbor a CXCR5+ CD8+ T cell subset. **(A)** Representative gating strategy employed to identify Ova-specific CXCR5+ T cells using fluorescence minus one (FMO) control. **(B, C)** Frequencies **(B)** and absolute numbers **(C)** of CXCR5+ Ova-specific CD8+ T cells in the liver of high antigen mice post AdOva vaccination (see **Figure S1B** for experimental details). At indicated time points after vaccination, liver non-parenchyma cells were isolated, and phenotyped for CXCR5+ Ova specific CD8+ T cells. Stained cells were analyzed by flow cytometry. **(D)** Frequency of Bcl6 expression in naïve, Ova- specific CXCR5+ and CXCR5- CD8+ T cells 21 days post vaccination. **(E)** MFI of Bcl6 expression in naïve, Ova-specific CXCR5+ and CXCR5- T cells in **(C)**. Representative histogram overlay (left) and summary of Bcl6 MFI (right). **(F)** Serum concentration of CXCL13 from AdOva vaccinated wildtype and high antigen in mice, as well as high antigen mice without vaccination. **(G)** Frequency of LFA-1 (CD11b) on CXCR5+ and CXCR5- CD8+ T cells 21 days post OT-1 transfer. **(H)** MFI of LFA-1 expression in **(G)**. Representative histogram plot (left) and summary (right) of LFA-1. **(I)** Frequency of Nur77 expression in CXCR5+ and CXCR5- Ova-specific CD8+ T cells. **(J)** MFI of Nur77 expression in naïve, CXCR5+ and CXCR5- CD8+ T cells. Representative histogram plot (left) and summary (right) of Nur77. *p ≤ 0.05; **p ≤ 0.01; ns: p > 0.05.

We asked whether Ova antigen recognition in the liver – a non-lymphoid organ – would facilitate the generation and retention of CXCR5+ CD8+ T cells in the liver, as previously demonstrated in the spleen after chronic LCMV infection (10, 11). Upon AdOva vaccination of high antigen mice, we profoundly observed ~20%–30% of Ova-specific CXCR5+ CD8+ T cells in the liver, which plateaued from D9 (**Figures 1A, B**). The absolute number of CXCR5+ CD8+ T cells were relatively maintained for at least 42 days (**Figure 1C**). To ascertain the identity of CXCR5+ CD8+ T cells in the liver we assessed Bcl6, a transcription factor that regulates CXCR5 expression. We observed that ~42%–66% of CXCR5+ CD8+ T cells expressed Bcl6 and the expression was largely enriched in CXCR5+ CD8+ T cells (**Figures 1D, E**, **S2A**). In contrast, Bcl6 expression in CXCR5- and endogenous naïve CD8+ T cells was comparable (**Figures 1D, E**, **S2A**). The presence of antigen specific T cells in liver was accompanied by increased serum concentrations of CXCL13 in AdOva vaccinated mice if compared to AdOva vaccinated wildtype mice (**Figure 1F**). Immune histological analysis further showed an increased frequency of CXCL13 expressing cells in liver (**Figure S1G**). These data, therefore, suggest that CXCL13 is induced upon antigen recognition in the liver and may foster the retention of CXCR5+ CD8+ T cells in the liver. We confirmed the tendency of CXCR5+ and CXCR5- Ova-specific CD8+ T cells to reside in the liver by assessing the canonical liver residency marker LFA-1 (34) as well as tissue resident markers CXCR6 and CX3CR1 (35–37). Of note, almost all the CXCR5+ and CXCR5- CD8+ T cell subsets expressed CX3CR1 and CXCR6 (**Figure S2B**), and LFA-1 was present on both subsets of T cells (**Figure 1G**, **S2C**). Instructively, we also observed higher LFA-1 median fluorescence intensity (MFI) on CXCR5+ CD8+ T cells (**Figure 1H**). LFA-1 has been shown to contribute to T cell proliferation and activation (38, 39). Therefore, we asked if CXCR5+ CD8+ T cells receive higher signaling events through their T cell receptor. In this regard, we quantified Nur77, a transcription factor that is upregulated in response to T cell receptor signaling. We observed a higher frequency of Nur77+ cells in the CXCR5+ compared to CXCR5- CD8+ T cell subset (**Figure 1I**, **S2D**). The assessment of Nur77 MFI showed enriched expression in CXCR5+ CD8+ T cells compared to CXCR5- CD8+ T cells as well as naïve CD8+ T cells (**Figure 1J**), indicating higher T cell receptor signaling strength in CXCR5+ CD8+ T cells. Collectively, these data suggest that CXCR5+ Ova-specific CD8+ T cells may be liver resident, exhibiting increased signaling through the T cell receptor.

### Intrahepatic CXCR5+ Ova-Specific CD8+ T Cells Show Enhanced Activation and Memory Properties Which Are Accompanied With Reduced Exhaustion

**Figure 2:**
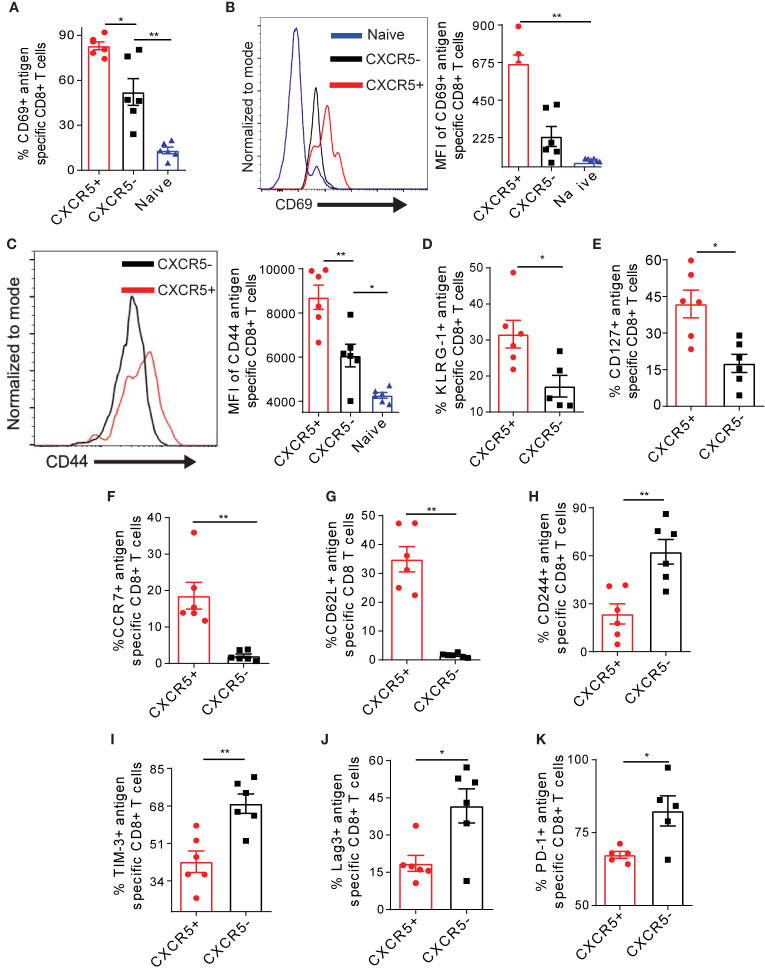
The CXCR5+ Ova-specific CD8+ T cell subset possesses enhanced activation and memory capacities coupled to reduced exhaustion. Liver non-parenchyma cells were isolated 21 days post AdOva vaccination of high antigen mice and phenotyped for Ova-specific CXCR5+ and CXCR5- CD8+ T cells expressing activation markers CD69, CD44 and KLRG-1 **(A–D)** memory markers CD62L, CCR7 and CD127 **(E–G)** and exhaustion markers CD244, TIM-3, PD-1 and Lag-3 **(H–K)**. Analysis was done by flow cytometry. **(A)** Frequency of CD69 expression on naïve, CXCR5+ and CXCR5- CD8+ T cells. **(B)** MFI of CD69 on naïve, Ova-specific CXCR5+ and CXCR5- CD8+ T cells in **(A)**. Representative histogram overlay (left) and summarized (right) MFI. **(C)** Summarized MFI of CD44 on CXCR5+ and CXCR5- CD8+ T cells. **(D)** Frequency of KLRG-1 on CXCR5+ and CXCR5- CD8+ T cell subsets. **(E)** Frequency of CD127+ CXCR5+ and CXCR5- CD8+ T cells. **(F)** Frequency of CCR7 on CXCR5+ and CXCR5- T cells. **(G)** Frequency of CD62L on CXCR5+ and CXCR5- CD8+ T cells. **(H)** Frequency of CD244 on CXCR5+ and CXCR5- CD8+ T cells. **(I)** Frequency of TIM-3 on CXCR5+ and CXCR5- CD8+ T cells **(J)** Frequency of Lag-3 on CXCR5+ and CXCR5- CD8+ T cells. **(K)** Frequency of PD-1 on CXCR5+ and CXCR5- CD8+ T cells. Data are representative of one out of two independent experiments. *p ≤ 0.05; **p ≤ 0.01.

We asked if the different level of Nur77 expression may be associated with distinct activation, exhaustion and memory characteristics of CXCR5+ and CXCR5- Ova-specific CD8+ T cells. We observed higher frequency and MFI of the activation marker CD69 on CXCR5+ CD8+ T cells on D21 post AdOva vaccination (**Figures 2A, B**, **S3A**). Although CXCR5+ and CXCR5- CD8+ T cell subsets expressed the activation marker CD44, CD44 MFI was significantly higher on CXCR5+ CD8+ T cells (**Figure 2C**). Likewise, the frequency of the activation marker KLRG-1 was higher in CXCR5+ CD8+ T cells (**Figure 2D**, **S3B**). Further, we probed the memory markers CD62L, CD127, and CCR7 expression on antigen specific CXCR5+ and CXCR5- CD8+ T cell subsets D21 post AdOva vaccination. Higher frequencies of CD127 and CCR7 were present on CXCR5+ CD8+ T cells (**Figures 2E, F**, **S3C, D**). In addition, we observed exclusive expression of CD62L on CXCR5+ CD8+ T cells (**Figures 2G**, **S3F**).

To evaluate the extent of exhaustion, we assessed the exhaustion markers CD244, TIM-3, Lag-3, and PD-1 on Ova- specific CXCR5+ and CXCR5- CD8+ T cells from the liver D21 post AdOva vaccination. We observed higher frequencies of CD244, Lag-3, TIM-3, and PD-1 on CXCR5- CD8+ T cells (**Figures 2H–K**, **S4A–D**). The MFIs of these exhaustion markers were not different between CXCR5+ and CXCR5- CD8+ T cells (**Figure S4E–H**). Also, the analysis of multiple exhaustion markers revealed a higher frequency of cells expressing both PD1 and Lag-3 as well as TIM-3 and CD244 in the CXCR5- CD8+ T cell population as opposed to the CXCR5+ CD8+ T cell population (**Figure S4I**). Putting together, these data indicate that in the liver microenvironment, the CXCR5+ Ova-specific CD8+ T cell subset possesses enhanced activation and memory properties, which is characterized by lower exhaustion marker expression.

### CXCR5 Expression Identifies a Subpopulation of Exhausted Ova-Specific CD8+ T Cells Exhibiting Residual Cytotoxic Function in the Liver

**Figure 3:**
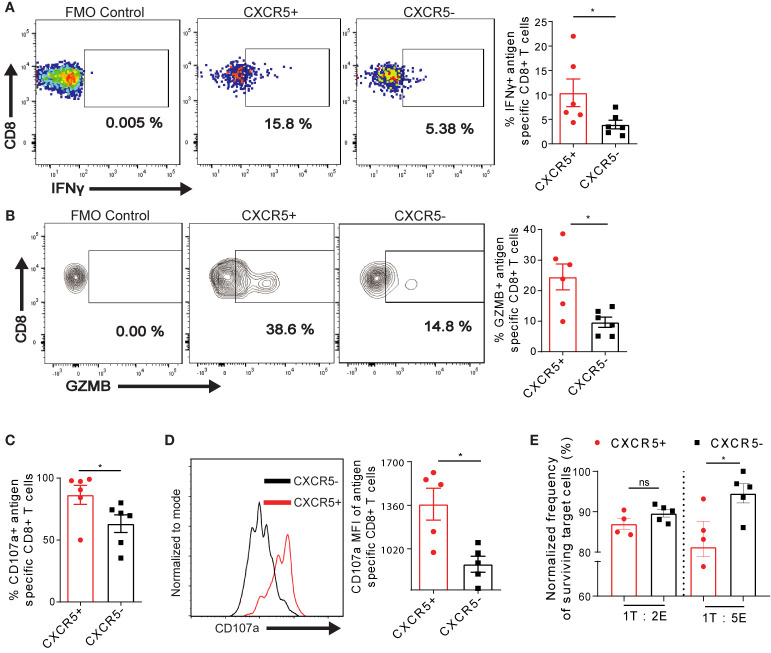
Residual cytotoxicity is enriched in exhausted Ova-specific CXCR5+ CD8+ T cells in the liver. **(A)** D21 post AdOva vaccination of high antigen mice, liver non-parenchyma cells were re-stimulated ex vivo with PMA/ionomycin for 4-5h. The cells were phenotyped for **(A)** IFN-λ and **(B)** GZMB in Ova-specific CXCR5+ and CXCR5- CD8+ T cells and data was analyzed by flow cytometry. Representative dot plots (left) and summary (right). **(C, D)** D21 post AdOva vaccination, liver non-parenchyma cells were re-stimulated ex vivo with PMA/ionomycin in the presence of anti-CD107a for 3-4h. The cells were phenotyped for Ova-specific CXCR5+ and CXCR5- CD8+ T cells, and data was analyzed by flow cytometry. **(C)** Frequency of CD107a in antigen specific CXCR5+ and CXCR5- CD8+ T cells. **(D)** MFI of CD107a in antigen specific CXCR5+ and CXCR5- CD8+ T cells. Representative histogram (left) and summary (right). **(E)** *In vitro* killing of Ova-peptide pulsed EL4 cells by CXCR5+ and CXCR5- OT-1 cells. D14 post adoptive transfer of dsRED+ OT-1 cells, dsRED+ OT-1 cells from the liver were sorted into CXCR5+ and CXCR5- CD8+ T cells. The CXCR5+ and CXCR5- CD8+ T cells were co-cultured with EL4 cells pulsed with Ova-peptide for 24h in the ratios of 1 Target (T) : 2 Effector (E) and 1T:5E. The pulsed EL4 target cells were normalized to un-pulsed EL4 cells to determine the rate of T cell induced EL4 killing. *p ≤ 0.05; ns: p > 0.05.

To address if the differential expression of exhaustion markers on intrahepatic T cells potentially affects T cell activity, we analyzed the frequency of effector cytokines IFN-γ in Ova-specific CXCR5+ and CXCR5- CD8+ T cells D21 post AdOva vaccination. CXCR5+ CD8+ T cells showed a minor increase in the frequency of IFN-γ expression (**Figure 3A**) as well a low but significant increase of IFN-γ MFI (**Figure S5A**). We thus asked if the CXCR5+ Ova-specific CD8+ T cells also upregulate the cytotoxic molecules GZMB and CD107a. Notably, we quantified a higher frequency of GZMB in CXCR5+ CD8+ T cells (**Figure 3B**). Moreover, further analysis showed that almost all CXCR5+ CD8+ T cells expressed CD107a compared to ca. 60% in CXCR5- CD8+ T cells (**Figure 3C**, **S5B**). In addition, the MFI of CD107a was elevated in the CXCR5+ CD8+ T cell compartment (**Figure 3D**).

We evaluated the consequences of the enriched expression of GZMB and CD107a in Ova-specific CXCR5+ CD8+ T cells compared to CXCR5- CD8+ T cells by determining the killing capacity of the cells *in vitro*. To this end, on D14 post transfer of naïve dsRED+ OT-1 cells into high antigen mice, CXCR5+ and CXCR5- OT-1 cells were sorted by flow cytometry, and co-cultured with OVA-peptide pulsed EL4 target cells. We injected mice intravenously with the s+16a nanobody, isolated cells from the liver and sorted for CXCR5+ and CXCR5- OT-1 cells by FACS. The s+16a nanobody preserves the vitality and promotes higher recovery of liver resident T cells (40, 41). We chose D14 post transfer because of the documented evidence that D3 post OT-1 transfer, the exhaustion trajectory has already been initiated (29). Effector-target cell ratios of 2:1 and 5:1 were exploited for this purpose. We observed enhanced killing of target EL4 cells by CXCR5+ OT-1 cells compared to CXCR5- OT-1 cells in both conditions, albeit to different degrees (**Figure 3E**). Taking together, these data demonstrate that Ova- specific CD8+ T cells responding to chronic Ova- antigen in the liver comprise CXCR5+ cells, which are less exhausted and possess enhanced residual cytotoxic functions.

### Exhausted CXCR5+ Ova-Specific CD8+ T Cells Demonstrate Efficient Mitochondria and Nutrient Acquisition Function

**Figure 4:**
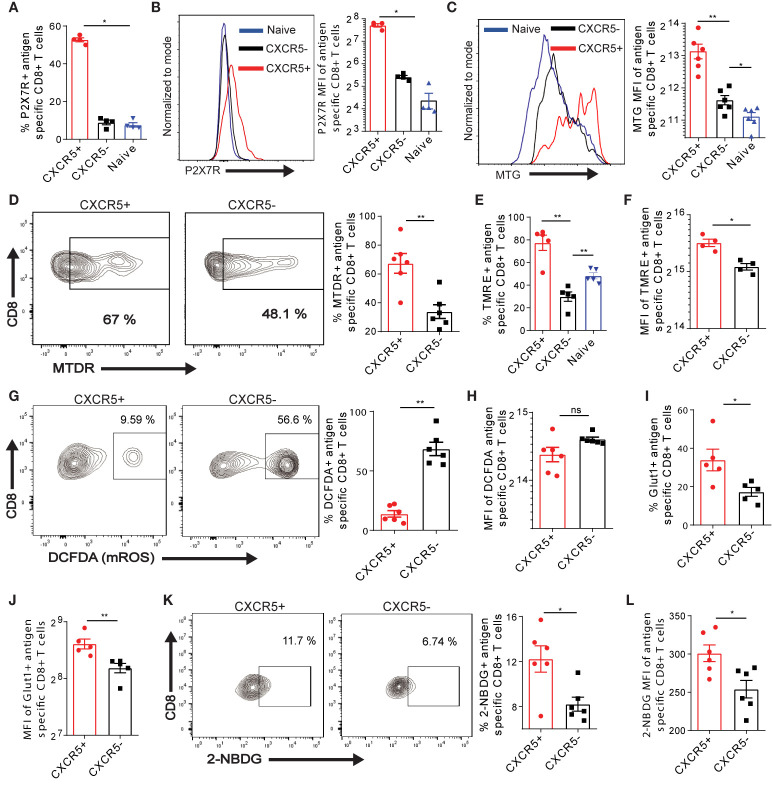
CXCR5 identifies exhausted Ova-specific CD8+ T cells with efficient mitochondria and nutrient acquisition function. **(A)** Frequency of the purinergic receptor P2X7 on naïve, CXCR5+ and CXCR5- CD8+ T cells. D21 post vaccination of high antigen mice, P2X7R was assessed on naïve, Ova-specific CXCR5+ and CXCR5- CD8+ T cells. Data was analyzed with flow cytometry. **(B)** MFI of P2X7R expression on naïve, CXCR5+ and CXCR5- CD8+ T cells in **(A)**. Representative histogram (left) and summarized MFI (right). **(C–I)** Liver non-parenchyma cells were isolated D21 post AdOva vaccination or adoptive transfer of OT-1 cells. The cells were stained with mitochondria sensitive dyes mitotracker green (MTG), deep red (MTDR), TMRE and mROS sensitive dye DCFDA and analyzed for Ova-specific CXCR5+ and CXCR5- CD8+ T cells by flow cytometry. The data shown are from AdOva vaccination experiments, OT-1 transfer showed similar outcome. **(C)** MTG MFI of CXCR5+ and CXCR5- CD8+ T cells. Representative histogram plot (left) and summary (right). **(D)** MTDR frequency of CXCR5+ and CXCR5- CD8+ T cells. Representative dot plots (left) and summary (right). **(E)** Frequency of TMRE in naïve, antigen specific CXCR5+ and CXCR5- CD8+ T cells. **(F)** MFI of TMRE in CXCR5+ and CXCR5- CD8+ T cells in **(E)**. **(G)** Frequency of DCFDA in CXCR5+ and CXCR5- CD8+ T cells. Representative dot plots (left) and summary (right). **(H)** MFI of DCFDA in CXCR5+ and CXCR5- CD8+ T cells in **(G)**. **(I)** Frequency of Glut-1 on CXCR5+ and CXCR5- CD8+ T cells. **(J)** MFI of Glut-1 expression in CXCR5+ and CXCR5- CD8+ T cells in **(I)**. **(K)** Frequency of 2-NBDG uptake in CXCR5+ and CXCR5- CD8+ T cells. Representative dot plots (left) and summary (right). **(L)** 2-NBDG MFI of CXCR5+ and CXCR5- CD8+ T cell subsets in **(K)**. **p ≤ 0.01; *p ≤ 0.05; ns p > 0.05.

The activation induced differentiation of T cells is, in part, profoundly regulated by the metabolism. A recent study suggested that the purinergic receptor P2X7R drives the metabolic fitness and long-term maintenance of CD8+ T cells in acute LCMV infection (42). We addressed the question of whether P2X7R is expressed on exhausted Ova-specific CD8+ T cells in the liver. Interestingly, we observed that P2X7R was mainly expressed on Ova-specific CXCR5+ CD8+ T cells as opposed to CXCR5- and naive CD8+ T cells (**Figure 4A**, **S6A**). Further, we quantified a higher P2X7R MFI on CXCR5+ CD8+ T cells (**Figure 4B**), suggesting that Ova-specific CXCR5+ CD8+ T cells may be exhausted but long-lived memory cells. The preferential expression of P2X7R on CXCR5+ CD8+ T cells (**Figures 4A, B**) coupled to enhanced memory properties (**Figure 2**), led to the hypothesis that efficient mitochondrial function may be restricted largely to the Ova-specific CXCR5+ CD8+ T cell subset. We tested this hypothesis by assessing different aspects of mitochondria function in Ova-specific CD8+ T cells, D21 post AdOva vaccination. Using Mitotracker Green dye (MTG) staining, we observed that the mass of the mitochondria was increased in Ova-specific CXCR5+ CD8+ T cells compared to the CXCR5- CD8+ T cell subset. More so, we observed slightly higher mitochondria mass in CXCR5- CD8+ T cells compared to naïve endogenous T cells (**Figure 4C**). Since memory T cells have higher mitochondria mass compared to effector T cells (43, 44), these data are in agreement with the enhanced memory marker expression (**Figure 2**). Subsequently, we quantified the mitochondria potential of Ova-specific CXCR5+ and CXCR5- CD8+ T cells, using Tetramethylrhodamine ethyl ester (TMRE) and Mitotracker Deep Red (MTDR). We observed a higher frequency of MTDR and TMRE in the CXCR5+ T cell compartment (**Figures 4D, E**, **S6B**). Moreover, the MFI of TMRE was reduced in CXCR5- CD8+ T cells (**Figure 4F**), suggesting reduced mitochondria membrane potential in this subset. Reduced mitochondria activity may be a result of excessive production of mitochondria reactive oxygen species (mROS), which disrupts mitochondria function (45). Upon probing mROS in antigen specific T cells, we observed ~60% of Ova-specific CXCR5- CD8+ T cells while a minority of CXCR5+ CD8+ T cells expressed mROS (**Figure 4G**). Interestingly, mROS MFI was higher in CXCR5- CD8+ T cells, albeit not significantly (**Figure 4H**).

Effector cytokine production is largely coupled to the bioenergetics and nutrient acquisition efficiency of T cells (46, 47). These processes are extensively and tightly regulated by the mitochondria function. Therefore, we asked if the efficient mitochondria function and the improved cytotoxic function may be associated with elevated nutrient acquisition in Ova-specific CXCR5+ T cells. In this regard, Ova-antigen specific T cells isolated from the liver D21 post AdOva vaccination were phenotyped for the glucose 1 transporter (Glut-1). We observed a higher frequency of Glut-1 expression in CXCR5+ CD8+ T cells compared to the CXCR5- CD8+ T cell subset (**Figure 4I**, **S6C**). Quantification of Glut-1 MFI further indicated higher protein levels in CXCR5+ CD8+ T cells (**Figure 4J**). To evaluate the functionality of Glut-1 expression by CXCR5+ and CXCR5- CD8+ T cells we determined the uptake of fluorescent glucose (2-NBDG). In concord to higher Glut-1 expression, higher frequency and MFI of 2-NBDG uptake were observed from Ova-specific CXCR5+ CD8+ T cells (**Figures 4K, L**). Collectively, these data suggest that improved mitochondria function of Ova-antigen specific CD8+ T cells in the liver is intimately linked to memory-like CXCR5+ CD8+ T cells, which exhibit efficient nutrient uptake.

### Exhausted CXCR5+ and CXCR5- Ova-Specific CD8+ T Cells Display Differential Maintenance and Proliferation

**Figure 5:**
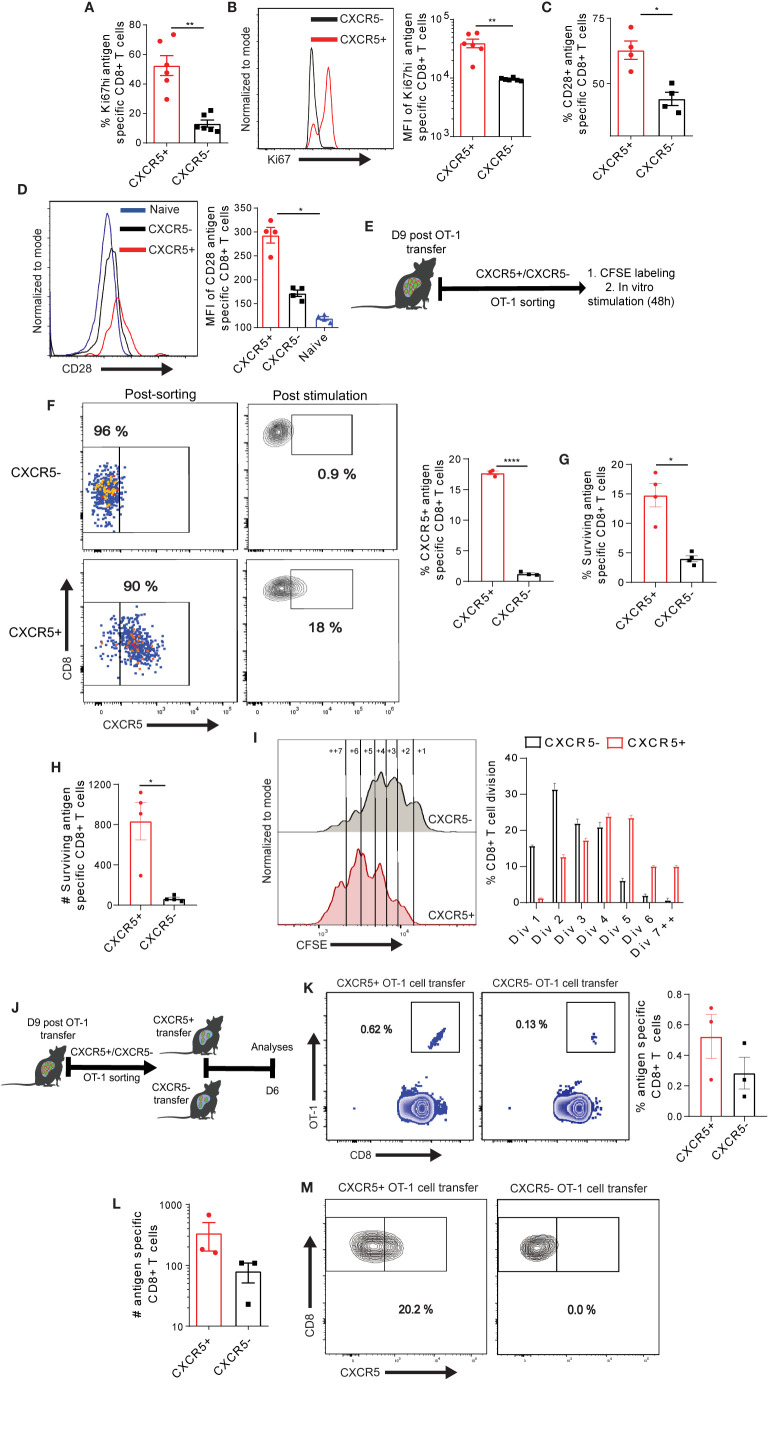
Differential maintenance and proliferation of exhausted CXCR5+ and CXCR5- Ova-specific CD8+ T cell subsets. **(A)** Frequency of Ki67hi in CXCR5+ and CXCR5- CD8+ T cells. D21 post AdOva vaccination of high antigen mice, Ova-specific CXCR5+ and CXCR5- CD8+ T cells were phenotyped for Ki67 expression. Data was analyzed by flow cytometry. **(B)** MFI of Ki67hi in CXCR5+ and CXCR5- CD8+ T cells in **(A)**. Representative histogram plot (left) and summary (right). **(C)** Frequency of CD28 on naïve, CXCR5+ and CXCR5- CD8+ T cells. D21 post AdOva vaccination, CD28 was analyzed on naïve, Ova-specific CXCR5+ and CXCR5- CD8+ T cells. **(D)** CD28 MFI of naïve, CXCR5+ and CXCR5- CD8+ T cell subset in **(C)**. Representative histogram plot (left) and summary (right). **(E–G)** D9 post transfer of naïve dsRED+ OT-1 cells into high antigen mice, CXCR5+ and CXCR5- OT-1 cells were FACS sorted. The sorted cells (CXCR5+ and CXCR5-) were CFSE labeled and stimulated in vitro for 48h. The cells were harvested, counted, phenotyped and analyzed by flow cytometry. **(E)** The experimental setup for **(F–M)**. **(F)** *In vitro* differentiation pattern of CXCR5+ and CXCR5- OT-1 cells after stimulation. Representative dot plots of FACS sorted cells and conversion of CXCR5+ and CXCR5- OT-1 cells (left), and summary (right). **(G)** Frequency of surviving CXCR5+ and CXCR5- OT-1 cells 48 h post stimulation. **(H)** Absolute number of surviving CXCR5+ and CXCR5- OT-1 cells in **(G)**. **(I)** CFSE dilution of CXCR5+ and CXCR5- OT-1 cells. Representative histogram plot showing the number of cell divisions (left) and summary of the frequency of OT-1 cells in each cell cycle (right). **(J–M)** D9 post naïve dsRED+ OT-1 transfer into high antigen mice, CXCR5+ and CXCR5- OT-1 cells were FACS sorted. The sorted CXCR5+ and CXCR5- OT-1 cells were transferred into low antigen recipient mice. D6 post transfer of CXCR5+ and CXCR5- OT-1 cells, non-parenchyma cells were isolated and analyzed for dsRED+OT-1 cells in the liver. **(J)** The experimental setup. **(K)** Representative dot plots (left) and summary (right) of antigen specific T cells in recipient mice. **(L)** Absolute numbers of OT-1 cells in the liver of CXCR5+ and CXCR5- recipient mice. **(M)** Representative dot plots of the differentiation of CXCR5+ and CXCR5- OT-1 cells. ****p ≤ 0.001; **p ≤ 0.01; *p ≤ 0.05.

We hypothesized that the improved metabolic properties of Ova-specific CXCR5+ CD8+ T cells may facilitate rapid proliferation and, possibly, other energy demanding cellular processes. Thus, we examined the cell cycle marker Ki67 expression in Ova-specific CD8+ T cells. We observed that all the Ova-specific CXCR5+ and CXCR5- CD8+ T cells were positive for Ki67, however, a careful examination showed a higher frequency of CXCR5+ CD8+ T cells expressing high levels of Ki67 (Ki67^hi^), and an increased MFI of Ki67 in CXCR5+ CD8+ T cell subset (**Figures 5A, B**, **S6C**). Next, we analyzed the expression of the co-stimulatory molecule CD28 within antigen specific CD8+ T cells. T cell receptor signaling alongside the co-stimulatory molecule CD28 has been shown to promote T cell function and proliferation (48, 49). Congruent to the increased Ki67 expression, we observed higher CD28 on CXCR5+ CD8+ T cells compared to CXCR5- CD8+ T cells (**Figure 5C**, **S6D**). Analysis of CD28 MFI showed 50% reduction on Ova-specific CXCR5- CD8+ T cells (**Figure 5D**). We asked if the expression levels of Ki67 and CD28 are functionally relevant for CXCR5+ CD8+ T cell proliferation. To test the proliferation, differentiation and maintenance capacity of Ova-specific CXCR5+ and CXCR5- CD8+ T cells, we transferred dsRED+ OT-I cells to high antigen mice (**Figure 5E**). On D9 post OT-1 transfer, mice were intravenously injected with s+16a nanobody prior to liver non-parenchyma cell isolation. The CXCR5+ and CXCR5- dsRED+ OT-1 cells were FACS sorted and labeled with the proliferation tracker dye CFSE (**Figure 5E**). D9 was selected because of the recovery of highest numbers of Ova-specific CD8+ T cells (**Figure S1D**). Upon *in vitro* stimulation with anti-CD3/CD28 coupled Dynabeads, the CXCR5- OT-1 cells remained largely CXCR5-. In contrast, the CXCR5+ OT-1 cells differentiated into CXCR5- OT-1 cells but also maintained a small fraction of CXCR5+ OT-1 cells (**Figure 5F**). Interestingly, we observed higher survival of CXCR5+ OT-1 cells upon stimulation (**Figures 5G, H**). Furthermore, we assessed the proliferation capacity by evaluating the dilution of CFSE in surviving CXCR5+ and CXCR5- OT-1 cells (**Figure 5I**). We observed a rather slow rate of cell division in CXCR5- OT-1 cells while the CXCR5+ OT-1 cells underwent rapid cell division (**Figure 5I**) as reflected by a higher frequency of CXCR5+ OT-1 cells in >4 cell division cycles. These data, therefore, suggest that CXCR5+ CD8+ T cells have a survival and proliferative advantage over CXCR5- CD8+ T cells, and CXCR5+ CD8+ T cells represent a progenitor T cell subset capable of generating CXCR5- CD8+ T cells.

To evaluate if the higher *in vitro* proliferation and survival properties are also displayed *in vivo*, we transferred naive dsRED+ OT-1 cells into high antigen mice (**Figure 5J**). D9 post T cell transfer, CXCR5+ and CXCR5- OT-1 cells were FACS sorted from liver post s+16a nanobody treatment and transferred into low antigen recipient mice (**Figure 5J**). D6 post CXCR5+ and CXCR5- OT-1 cell transfer, non-parenchyma cells were isolated from liver and analyzed by flow cytometry for dsRED+ OT-1 cells. Although not statistically significant, we observed higher frequencies and absolute numbers of dsRED+OT-1 cells in the liver of CXCR5+ OT-1 cell recipient mice compared to the CXCR5- OT-1 cell recipient mice (**Figures 5K, L**). In agreement with the *in vitro* analysis, CXCR5- OT-1 cell remained CXCR5- while the CXCR5+ OT-1 cells partially differentiated to CXCR5- OT-1 cells (**Figure 5M**). In sum, these data suggest that Ova-specific CXCR5+ CD8+ T cells have a proliferation, self-maintenance and memory recall advantage over the CXCR5- CD8+ T cell subset in the liver.

### Host Conditioning With CpG ODN Reinvigorates Exhausted Ova-Specific CD8+ T Cells and Promotes Intrahepatic T Cell Formation

**Figure 6:**
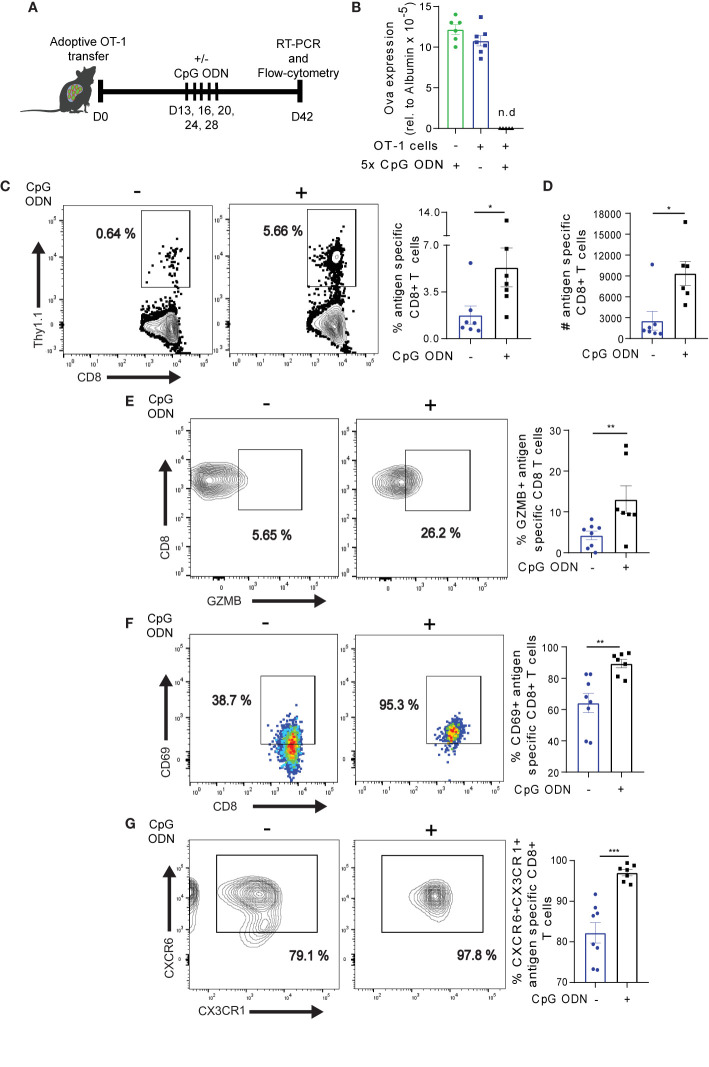
CpG ODN-mediated reinvigoration of exhausted Ova specific CD8+ T cells promotes liver resident T cell formation. **(A)** The experimental set-up for Fig 6 and Fig. 7. 3-5 x 10^6^ OT-1 cells were adoptively infused into high antigen mice. D13-28 post OT-1 transfer, mice were treated 5x with CpG ODN or mock as indicated. On D42, Ova specific T cells were analyzed by flow cytometry. The Ova transcript level in hepatocytes was determined by qRT-PCR. **(B)** Relative Ova expression from the liver of high antigen mice with (blue bar) or without (green bar) OT-1 transfer, and with OT-1 transfer plus CpG ODN (black bar) treatment. RNA was isolated from the liver of the respective condition and Ova levels were assessed by qRT-PCR analysis. The quantified Ova expression was normalized to hepatocyte specific (albumin) housekeeping gene. n.d.: not detected. **(C)** Frequency of Ova-specific CD8+ T cells in the liver with and without CpG ODN treatment. Representative dot plots (left) and summary (right). Liver non-parenchyma cells were isolated D42 post adoptive OT-1 transfer and phenotyped for antigen specific CD8+ T cells. **(D)** Absolute numbers of antigen specific CD8+ T cells in **(C)**. **(E)** Frequency of GZMB expression with and without CpG ODN conditioning. Representative dot plots (left) and summary (right) of GZMB. **(F)** Frequency of CD69 expression. Representative dot plots (left) and summary (right) of CD69. **(G)** Frequency of CXCR6+CX3CR1+ on antigen specific T cells. Representative dot plot (left) and summary (right). ***p ≤ 0.001; **p ≤ 0.01; *p ≤ 0.05.

We previously reported that host conditioning with CpG ODN during the onset of Ova-specific CD8+ T cell response – i.e., prior to the development of T cell exhaustion – precludes the formation of an exhausted phenotype (30). We asked if host conditioning with CpG ODN also has a beneficial effect on exhausted Ova-specific CD8+ T cells in the liver. To this end, we transferred naïve OT-1 cells into high antigen mice. On day 13 post adoptive OT-1 transfer, when T cells are exhausted (29), we treated the mice with CpG ODN over a period of 15 days (**Figure 6A**). On day 14 post the last CpG ODN application (D42 post adoptive transfer of cells), the mice were sacrificed and the antigen load in liver was determined. Strikingly, CpG ODN treated mice showed efficient antigen clearance from the liver (**Figure 6B**). Contrarily, control groups that received OT-1 cells without CpG ODN or CpG ODN without OT-1 cells did not clear the antigen load and showed Ova levels comparable to high antigen mice (**Figure 6B**). These data suggest that CpG ODN mediates the reprogramming of exhausted OT-1 cells, resulting in improved cytotoxic activity. To understand how CpG ODN mediated the reprogramming of exhausted Ova-antigen specific T cells on the cellular level, we analyzed the OT-1 cells in the liver of CpG ODN treated mice. We observed a significant increase in the frequency and absolute number of OT-1 cells upon CpG ODN conditioning (**Figures 6C, D**), suggesting improved maintenance of antigen specific CD8+ T cells. Moreover, the frequency of GZMB producing OT-1 cells was enriched in CpG ODN recipient mice (**Figure 6E**), suggesting that the functionality of T cells was improved. Interestingly, we observed higher levels of the activation marker CD69 expression on OT-1 cells in CpG ODN treatment (**Figure 6F** **).** T cells expressing CD69 in the absence of antigen were previously shown to possess improved tissue residency capacity (50, 51). To determine whether in our model the CD69+ CD8+ T cells express other markers of tissue resident memory T cells, we stained for CXCR6 and CX3CR1. Of note, OT-1 cells isolated from the group of high antigen mice treated with CpG ODN homogeneously expressed CXCR6 and CX3CR1 as opposed to OT-1 cells from control mice without CpG ODN treatment (**Figure 6G**), suggesting that CD69+ OT-1 cells after antigen clearance are possibly tissue resident T cells. Taking together, these data suggest that CpG ODN may: (a) rewire exhausted OT-1 cell function and/or (b) induce extensive proliferation in the less exhausted CXCR5+ T cells to support cytotoxicity and promote intrahepatic memory T cell formation.

### CpG ODN-Mediated Reinvigoration of Exhausted Ova-Specific T Cells Potentiates the Maintenance of Long-Lived CXCR5+ CD8+ T Cells

**Figure 7:**
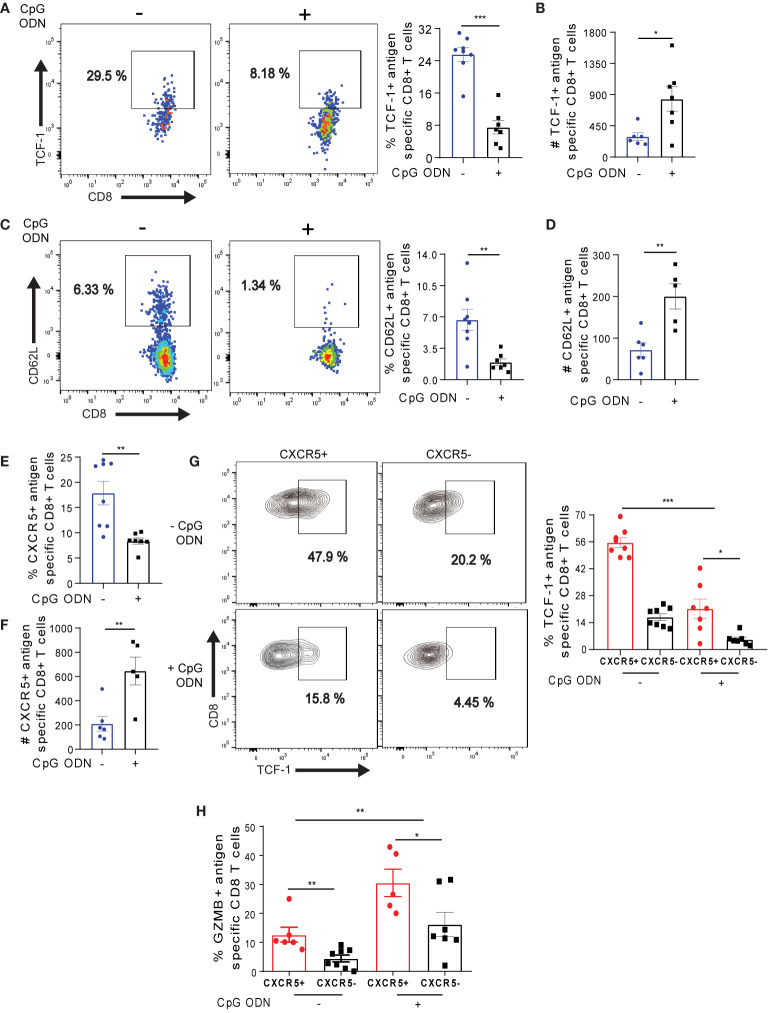
CpG ODN-mediated host conditioning promotes the maintenance of long-lived Ova-specific CXCR5+ CD8+ T cells. **(A–G)** On D42 post OT-1 transfer, OT-1 cells isolated from CpG ODN and non-CpG treatment (see **Figure 6A** for experimental setup) were phenotyped for flow cytometry analysis. **(A)** Frequency of TCF-1 expression on OT-1 cells on D42. Representative dot plots (left) and summary (right). **(B)** Number of TCF-1+ OT-1 cells in **(A)**. **(C)** Frequency of CD62L+ OT-1 cells. Representative dot plots (left) and summary (right). **(D)** Absolute number of CD62L expressing OT-1 cells. **(E)** Frequency of CXCR5+ OT-1 cells. **(F)** Absolute number of CXCR5+ OT-1 cells in **(E)**. **(G)** Frequency of TCF-1 in CXCR5+ and CXCR5- OT-1 cells with or without CpG ODN treatment. Representative dot plots (left) and summary (right). **(H)** Frequency of GZMB expressing CXCR5+ and CXCR5- OT-1 cells with or without CpG ODN treatment. On D42 post OT-1 transfer, liver non-parenchyma cells were ex-vivo re-stimulated for 4h, phenotyped and data was analyzed by flow cytometry. ***p ≤ 0.001; **p ≤ 0.01; *p ≤ 0.05.

To determine how CpG ODN mediated reprogramming affects exhausted Ova-specific T cell subsets, we first assessed the transcription factor TCF-1, which regulates the maintenance of T cells (52) and the memory marker CD62L on antigen specific T cells (37). OT-1 cells from CpG ODN treated mice effectively down-regulated TCF-1 and CD62L expression (**Figures 7A, C**), suggesting that CpG ODN potentiates the reduced expression of TCF-1 and CD62L in OT-1 cells. Still, the absolute numbers of TCF-1 and CD62L expressing cells in the pool of OT-1 cells were significantly higher (**Figures 7B, D**). While the frequency of CXCR5+ OT-1 cells was reduced (**Figure 7E**), notably, the absolute numbers of CXCR5+ OT-1 cells were increased upon CpG ODN treatment (**Figure 7F**) which is in agreement with the TCF-1 and CD62L analysis. Further, we assessed the expression of TCF-1 in CXCR5+ and CXCR5- OT-1 cell subsets in the absence of CpG ODN treatment. We observed maximum TCF-1 expression in the CXCR5+ OT-1 cell subsets (**Figure 7G**), which is in accordance to the previous notion that TCF-1 may potentially drive the long term-survival of antigen specific CXCR5+ CD8+ T cells (14, 15).

We further asked if the CpG ODN treatment would affect the expression of TCF-1 in CXCR5+ OT-1 cells. As expected, we observed a decreased frequency of TCF-1 in both CXCR5+ and CXCR5- OT-1 cells after CpG ODN conditioning (**Figure 7G**), suggesting that the inflammatory milieu created by CpG ODN may be involved in dampening TCF-1 expression as previously shown in context of vaccination or *L. monocytogenes* infection (53). Importantly, the frequency of TCF-1 expression was increased in CXCR5+ OT-1 cells both with or without CpG ODN conditioning (**Figure 7G**). Finally, we assessed the impact of CpG ODN treatment on GZMB expression in antigen specific CXCR5+ and CXCR5- OT-1 cells. With CpG ODN treatment, we observed 2-fold increased levels of GZMB expression in both CXCR5+ and CXCR5- OT-1 cells (**Figure 7H**). Taking together, these data suggest that although host conditioning with CpG ODN drives the depletion of TCF-1 in Ova-specific CXCR5+ CD8+ T cells, it promotes the maintenance of long-lived memory T cells and potentiates the enrichment of GZMB in both CXCR5+ and CXCR5- Ova-specific CD8+ T cells.

## Discussion

The *in-situ* regulation of exhausted T cells in chronic liver infections remains unclear in many aspects, largely due to the lack of small animal models which would recapitulate HBV and HCV infection as well as the respective consequences including liver fibrosis, liver cancer, and liver failure. In recent chronic LCMV infection studies, lymphoid associated CXCR5+ CD8+ T cells were shown to facilitate the long-term maintenance of exhausted T cells, providing the basis for PD-1 mediated reinvigoration of exhausted T cells (10, 11). Although previous studies gave valuable insights (54–56), the dynamic in-situ function of CXCR5+ CD8+ T cells in chronic HBV and HCV infection remains to be fully explored. In this study, we have identified a canonical CXCR5+ CD8+ T cell subset within the pool of exhausted Ova-specific T cells responding to persistent Ova antigen in the liver. We observed that the frequencies and numbers of CXCR5+ CD8+ T cells are relatively stable in the presence of persistent antigen. Effectively, CXCR5+ CD8+ T cells show potent cytotoxic function, possibly by virtue of their reduced exhaustion state coupled to improved T cell receptor signaling. Although cytotoxic, the Ova-specific CXCR5+ CD8+ T cells are memory-like with enhanced mitochondria function and nutrient uptake potential, suggesting they may utilize oxidative phosphorylation (OXPHOS) and glycolysis for energy metabolism. Importantly, we observed a rapid proliferation burst in CXCR5+ CD8+ T cells *in vitro*, and a better recall response to cognate antigen *in vivo*. The CXCR5+ CD8+ T cells from the liver maintain themselves and generate CXCR5- CD8+ T cells whereas the CXCR5- CD8+ T cells lack the ability to generate CXCR5+ CD8+ T cells. Importantly, mice conditioning with CpG ODN reinvigorates the exhausted Ova-specific CD8+ T cell pool in the liver, potentiating persistent antigen eradication from the liver. Upon reinvigoration, we observed higher numbers of Ova-specific CD8+ T cells and an increased number of resident memory T cells in the liver.

The hierarchical differentiation of T cells during chronic viral infection fosters heterogeneity, which drives the long-term maintenance of exhausted T cells (3, 4). This heterogeneity is reflected by progenitor CXCR5+ and terminally exhausted CXCR5- CD8+ T cell subsets (3, 33). The progenitor CXCR5+ CD8+ T cells were initially identified in context of LCMV infection and were reported to localize exclusively in lymphoid organs (10, 57). However, we have herein identified memory-like CXCR5+ Ova-specific CD8+ T cells in the liver – the site of antigen recognition. This suggests that the site of antigen recognition may be the fate determinant of CXCR5+ CD8+ T cells’ tissue localization. The CXCR5 chemoattractant CXCL13 is enriched in lymphoid organs (16), suggesting that the liver is not the natural homeostatic niche for CXCR5+ CD8+ T cells. Of note, a recent study documented that high levels of CXCL13 in livers of chronically infected HBV patients facilitate intrahepatic recruitment of CXCR5+ CD8+ T cells (54). In the study herein, we show that at the onset of antigen recognition, CXCL13 levels increase in serum. While the particular cell type expressing CXCL13 in course of intrahepatic T cell responses remains to be elucidated, our results indicate that CXCL13 may co-operate with antigen recognition to facilitate the retention of Ova-specific CXCR5+ CD8+ T cells in the liver. The retained CXCR5+ CD8+ T cells in the liver were relatively stable over time, indicating that the CXCR5+ CD8+ T cells may undergo constitutive self-maintenance and renewal, as previously suggested (11). Notably, upon transplantation into low antigen recipient mice the CXCR5+ CD8+ T cells largely differentiate to CXCR5- CD8+ T cells, while a small fraction of CXCR5+ CD8+ T cells was maintained. Therefore, the CXCR5+ CD8+ T cells may be referred to as a progenitor exhausted Ova-specific T cell subset in the liver.

The upregulation of LFA-1 has been shown to foster the retention of T cells in the liver (34). Likewise, the expression of CXCR6 and CX3CR1 (35–37) is also known to identify tissue resident T cells. We observed the expression of CXCR6, CX3CR1, and LFA-1 on Ova-specific CXCR5+ and CXCR5- CD8+ T cells, suggesting that these may be tissue resident cells. Tissue residency of T cells is regulated by complex transcriptional signatures (58, 59). Accordingly, the expression of these surface markers may not be sufficient to define the intrahepatic T cells as tissue resident. Thus, detailed transcriptional profiling as well as experimental parabiosis studies would be required to fully confirm their tissue residency.

The strength of T cell receptor signaling is a rheostat for Nur77 expression (60) and signaling through the T cell receptor is shown to be reduced in exhausted T cells (8). Thus, the level of Nur77 expression herein may demonstrate the degree of exhaustion at play in T cells. Indeed, we observed reduced frequencies of exhaustion markers on CXCR5+ CD8+ T cells, coupled to higher Nur77 expression, which is indicative of a dampened state of exhaustion. Accordingly, CXCR5+ CD8+ T cells were more cytolytic. CXCR5+ CD8+ T cells have been previously shown to provide the proliferative burst upon PD-1 blockade therapy (10). Herein, we have demonstrated that upon *in vitro* stimulation and adoptive transfer, intrahepatic Ova-specific CXCR5+ CD8+ T cells underwent enhanced proliferation. These data suggest that in chronic liver infection, PD-1 therapy may preferably target and foster CXCR5+ CD8+ T cell proliferation and function to enhance the functionality of the pool of exhausted CD8+ T cells, and thereby facilitate disease remission.

The results obtained in our OVA model mice are in agreement with a recent study that was based on an AAV-HBV mouse model (54). Upon hydrodynamic injection of recombinant adeno-associated viral vectors encoding the HBV 1.2 replicon, CXCR5+ CD8+ T cells were observed in liver as well as in spleen and blood. Consistent with the OVA model herein, antigen specific CXCR5+ CD8+ T cells from the AAV-HBV model exhibited improved cytotoxic functions. The findings obtained from these independent mouse models hint towards a crucial role of these cells for a potent immune response in liver.

The extensive proliferation of the progenitor CXCR5+ CD8+ T cells coupled to the upregulation of Nur77 strongly suggest that they may preferably be amenable to immunomodulation. Indeed, CpG ODN reinvigorated exhausted T cells, with an enhanced GZMB expression in CXCR5+ CD8+ T cells, which highlights the notion that exhausted Ova-specific CD8+ T cells are not anergic. The CpG ODN mediated reinvigoration translates into enrichment of Ova-specific CD8+ T cells in the liver that consist of higher numbers of progenitor CXCR5+ CD8+ T cells. Interestingly, CpG ODN treatment downmodulates the expression of TCF-1 in CXCR5+ CD8+ T cells. This observation indicates that CpG ODN may drive the dampening of TCF-1, which controls memory T cell formation. Thus, CpG ODN may facilitate effector T cell differentiation. Indeed, a recent study showed that inflammatory cytokines suppress TCF-1 in T cells and promote the formation of effector cells (53). CpG ODN is known to mediate the upregulation of inflammatory cytokines and co-stimulatory molecules CD80/86 and OX40 on myeloid cells, which promotes improved T cell function (9). Signaling through OX40L and CD28, the receptor for CD80/86, facilitates enhanced proliferation of T cells (61). Therefore, exhausted Ova-specific T cell reinvigoration may partly be initiated by co-stimulatory signaling, which facilitates exhausted T cell proliferation and the recovery of high Ova-specific T cells. In fact, the success of PD-1 immunotherapy in chronic infection and cancer is dependent on CD28 signaling (62). Thus, CpG ODN treatment may preferentially induce extensive proliferation in CXCR5+ CD8+ T cells, as they mainly expressed CD28, which possibly promoted the rapid proliferation of CXCR5+ CD8+ T cells *in vitro*. Together, our data are in line with the hypothesis that CpG ODN reprogram exhausted T cells, thereby reinvigorating their cytotoxic capacity. In addition, the reinvigoration of exhausted T cells could be a result of targeted CpG ODN-mediated proliferation of the less exhausted and more functional CXCR5+ CD8+ T cells. Future studies would be needed to decipher the underlying mechanism facilitating CpG mediated exhausted CD8+ T cell revival.

The stemness properties of exhausted T cells are maintained by Bcl6 and TCF-1 co-operation *via* restraining type I interferon pro-exhaustion mechanisms (15). In addition, the regulation of exhausted T cell stemness is mediated by a complex transcriptional network, with TCF-1 playing a cardinal role (63). The restriction of Bcl6 and TCF-1 to CXCR5+ CD8+ T cells suggests that the TCF-1-Bcl6 axis may play a critical role in regulating the multi-potency, cytotoxicity, pro-survival and exhaustion states of CXCR5+ CD8+ T cells. Consistently, CXCR5+ CD8+ T cells are more cytotoxic and upregulate multiple memory associated markers. Of interest is the enriched expression of CD127 and the purinergic receptor P2X7R, which may predispose CXCR5+ CD8+ T cells to long-term survival (64, 65). Accordingly, CXCR5+ CD8+ T cells show better recall response and maintenance *in vitro* and *in vivo*.

The improved recall response may be intimately linked to the rapid proliferation and efficient mitochondria function of CXCR5+ Ova-specific CD8+ T cells. P2X7R was recently implicated in CD8+ T cell metabolism and this is mediated mechanistically *via* AMPK stimulation, OXPHOS, increased glucose and fatty acid uptake (42). Fused and elongated mitochondria networks promotes efficient electron transport chain activity, facilitating robust OXPHOS (66, 67). The mitochondria mass, which shapes memory T cell development (68), is larger in the CXCR5+ CD8+ T cells, suggesting efficient electron transport chain activity which may foster enhanced OXPHOS. Efficient electron transport chain activity regulates mROS expression (66, 68), which may compromise mitochondria integrity (69, 70). In this regard, the fragmented mitochondria in CXCR5- CD8+ T cells may be driving increased mROS, resulting in reduced mitochondria potential. Moreover, upregulation of Bcl6 dampens glycolysis (66), supporting the possible use of OXPHOS by CXCR5+ CD8+ T cells. Importantly, OXPHOS promotes increased spare respiratory activity, the ability to produce energy under stressful conditions (66, 67) – a hallmark of memory cells. Aside OXPHOS, CXCR5+ Ova-specific CD8+ T cells upregulate glucose intake, possibly to fuel glycolysis needed for rapid proliferation and cytotoxic function. Indeed, glucose is fed into the glycolytic pathway to fuel the cellular processes of T cells (66, 67). Therefore, we propose that CXCR5+ Ova-specific CD8+ T cells may depend on OXPHOS and glycolysis, which may be a unique feature of tissue resident memory T cells. These features support the bifunctional memory and cytotoxic nature of CXCR5+ CD8+ T cells.

In summary, based on the OVA mouse model that reflects the early stages of chronic hepatotropic viral infection, we have shown that the intrahepatic pool of exhausted Ova-specific CD8+ T cells consists of a specialized subpopulation of CXCR5+ CD8+ T cells. These CXCR5+ CD8+ T cells retain improved cytotoxic, metabolic, and proliferative functions upon chronic antigen stimulation. Accordingly, CXCR5+ CD8+ T cells may provide a novel option for therapeutic strategies to chronic liver infections in humans. However, experimental models that mimic the unique immunological characteristics upon HBV and HCV infection as well as the development of late stage liver diseases, would greatly facilitate our understanding of CXCR5+ CD8+ T cells for the treatment of infected patients. Of note, recent emerging strategies eliminating mouse-specific HCV restriction factors (19) might pave the way towards the development of such models.

## Data Availability Statement

The original contributions presented in the study are included in the article/**Supplementary Material**. Further inquiries can be directed to the corresponding authors.

## Ethics Statement

The animal study was reviewed and approved by Niedersächsisches Landesamt für Verbraucherschutz und Lebensmittelsicherheit, Dezernat 33. Written informed consent was obtained from the owners for the participation of their animals in this study.

## Author Contributions

Concept and design: KK and DW. Experiments and procedures: KK, MCP. Contribution of relevant resources: BR, FKN, CH, FK. All authors contributed to data analysis and interpretation of data. Writing the manuscript draft: KK, MC, BR, DW. All authors contributed to the article and approved the submitted version.

## Funding

This work was supported by grants from Deutsche Forschungsgemeinschaft (DFG) via the Cluster of Excellence ECX62 (REBIRTH – From Regenerative Biology to Reconstructive Therapy).

## Conflict of Interest

The authors declare that the research was conducted in the absence of any commercial or financial relationships that could be construed as a potential conflict of interest.

## References

[1] Cui W Kaech SM Generation of effector CD8+ T cells and their conversion to memory T cells. Immunol Rev (2010) 236:151–66. 10.1111/j.1600-065X.2010.00926.x PMC4380273 20636815

[2] Masopust D Schenkel JM The integration of T cell migration, differentiation and function. Nat Rev Immunol (2013) 13:309–20. 10.1038/nri3442 23598650

[3] McLane LM Abdel-Hakeem MS Wherry EJ CD8 T Cell Exhaustion During Chronic Viral Infection and Cancer. Annu Rev Immunol (2019) 37:457–95. 10.1146/annurev-immunol-041015-055318 30676822

[4] Hashimoto M Kamphorst AO Im SJ Kissick HT Pillai RN Ramalingam SS CD8 T Cell Exhaustion in Chronic Infection and Cancer: Opportunities for Interventions. Annu Rev Med (2018) 69:301–18. 10.1146/annurev-med-012017-043208 29414259

[5] Wherry EJ T cell exhaustion. Nat Immunol (2011) 12:492–9. 10.1038/ni.2035 21739672

[6] Sen DR Kaminski J Barnitz RA Kurachi M Gerdemann U Yates KB The epigenetic landscape of T cell exhaustion. Science (80-) (2016) 0491:1165–9. 10.1126/science.aae0491 PMC5497589 27789799

[7] Pauken KE Sammons MA Odorizzi PM Manne S Godec J Khan O Epigenetic stability of exhausted T cells limits durability of reinvigoration by PD-1 blockade. Science (80- ) (2016) 354:1160–5. 10.1126/science.aaf2807 PMC5484795 27789795

[8] Wherry EJ Kurachi M Molecular and cellular insights into T cell exhaustion. Nat Rev Immunol (2015) 15:486–99. 10.1038/nri3862 PMC4889009 26205583

[9] Huang L-RR Wohlleber D Reisinger F Jenne CN Cheng R-LL Abdullah Z Intrahepatic myeloid-cell aggregates enable local proliferation of CD8(+) T cells and successful immunotherapy against chronic viral liver infection. Nat Immunol (2013) 14:1–12. 10.1038/ni.2573 23584070

[10] Im SJ Hashimoto M Gerner MY Lee JJ Kissick HT Burger MC Defining CD8 + T cells that provide the proliferative burst after PD-1 therapy. Nature (2016) 537:417–21. 10.1038/nature19330 PMC5297183 27501248

[11] He R Hou S Liu C Zhang A Bai Q Han M Follicular CXCR5-expressing CD8(+) T cells curtail chronic viral infection. Nature (2016) 537:1–20. 10.1038/nature19317 27501245

[12] Mylvaganam GH Rios D Abdelaal HM Iyer S Tharp G Mavinger M Dynamics of SIV-specific CXCR5+ CD8 T cells during chronic SIV infection. Proc Natl Acad Sci (2017) 114:1976–81. 10.1073/pnas.1621418114 PMC5338410 28159893

[13] Chu F Li HS Liu X Cao J Ma W Ma Y CXCR5 + CD8 + T cells are a distinct functional subset with an antitumor activity. Leukemia (2019) 33(11):2640–53. 10.1038/s41375-019-0464-2 PMC6814517 31028278

[14] Wu T Shin HM Moseman EA Ji Y Huang B Harly C TCF1 Is Required for the T Follicular Helper Cell Response to Viral Infection. Cell Rep (2015) 12:2099–110. 10.1016/j.celrep.2015.08.049 PMC4591235 26365183

[15] Wu T Ji Y Moseman EA Xu HC Manglani M Kirby M The TCF1-Bcl6 axis counteracts type I interferon to repress exhaustion and maintain T cell stemness. Science Immunology (2016) 331(6):54–60. 10.1002/dev.21214.Developmental PMC5179228 28018990

[16] Shi J Hou S Fang Q Liu XX Liu XX Qi H PD-1 Controls Follicular T Helper Cell Positioning and Function. Immunity (2018) 49:264–74.e4. 10.1016/j.immuni.2018.06.012 30076099 PMC6104813

[17] Maini MK Burton AR Restoring, releasing or replacing adaptive immunity in chronic hepatitis B. Nat Rev Gastroenterol Hepatol (2019) 16:662–75. 10.1038/s41575-019-0196-9 31548710

[18] Dorner M Horwitz JA Robbins JB Barry WT Feng Q Mu K A genetically humanized mouse model for hepatitis C virus infection. Nature (2011) 474:208–12. 10.1038/nature10168 PMC3159410 21654804

[19] Brown RJP Tegtmeyer B Sheldon J Khera T Anggakusuma Todt D Liver-expressed *Cd302* and *Cr1l* limit hepatitis C virus cross-species transmission to mice. Sci Adv (2020) 6:eabd3233. 10.1126/sciadv.abd3233 33148654 PMC7673688

[20] Racanelli V Rehermann B The liver as an immunological organ. Hepatology (2006) 43(2 Suppl 1):54–62. 10.1002/hep.21060 16447271

[21] Crispe IN The liver as a lymphoid organ. Annu Rev Immunol (2009) 27:147–63. 10.1146/annurev.immunol.021908.132629 19302037

[22] Protzer U Maini MK Knolle PA Living in the liver: hepatic infections. Nat Rev Immunol (2012) 12:201–13. 10.1038/nri3169 22362353

[23] Sandhu U Cebula M Behme S Riemer P Wodarczyk C Metzger D Strict control of transgene expression in a mouse model for sensitive biological applications based on RMCE compatible ES cells. Nucleic Acids Res (2011) 39:1–13. 10.1093/nar/gkq868 20935052 PMC3017619

[24] Cebula M Ochel A Hillebrand U Pils MC Schirmbeck R Hauser H An Inducible Transgenic Mouse Model for Immune Mediated Hepatitis Showing Clearance of Antigen Expressing Hepatocytes by CD8+ T Cells. PLoS One (2013) 8:1–9. 10.1371/journal.pone.0068720 PMC3711822 23869228

[25] Aghajanian H Kimura T Rurik JG Hancock AS Leibowitz MS Li L Targeting cardiac fibrosis with engineered T cells. Nature (2019) 573:430–3. 10.1038/s41586-019-1546-z PMC6752964 31511695

[26] Ziegler PK Bollrath J Pallangyo CK Matsutani T Canli Ö De Oliveira T Mitophagy in Intestinal Epithelial Cells Triggers Adaptive Immunity during Tumorigenesis. Cell (2018) 174:88–101.e16. 10.1016/j.cell.2018.05.028 29909986 PMC6354256

[27] Riehn M Cebula M Hauser H Wirth D CpG-ODN facilitates effective intratracheal immunization and recall of memory against neoantigen-expressing alveolar cells. Front Immunol (2017) 8:1201. 10.3389/fimmu.2017.01201 29038654 PMC5630691

[28] Strandt H Pinheiro DF Kaplan DH Wirth D Gratz IK Hammerl P Neoantigen Expression in Steady-State Langerhans Cells Induces CTL Tolerance. J Immunol (2017) 199:1626–34. 10.4049/jimmunol.1602098 PMC5563165 28739880

[29] Ochel A Cebula M Riehn M Hillebrand U Lipps C Schirmbeck R Effective intrahepatic CD8+ T-cell immune responses are induced by low but not high numbers of antigen-expressing hepatocytes. Cell Mol Immunol (2015) 13(6):805–15. 10.1038/cmi.2015.80 PMC5101449 26412123

[30] Cebula M Riehn M Hillebrand U Kratzer RF Kreppel F Koutsoumpli G TLR9-Mediated Conditioning of Liver Environment Is Essential for Successful Intrahepatic Immunotherapy and Effective Memory Recall. Mol Ther (2017) 25:2289–98. 10.1016/j.ymthe.2017.06.018 PMC5628774 28716576

[31] Schirmbeck R Reimann J Kochanek S Kreppel F The immunogenicity of adenovirus vectors limits the multispecificity of CD8 T-cell responses to vector-encoded transgenic antigens. Mol Ther (2008) 16:1609–16. 10.1038/mt.2008.141 18612271

[32] Wortmann A Vöhringer S Engler T Corjon S Schirmbeck R Reimann J Fully detargeted polyethylene glycol-coated adenovirus vectors are potent genetic vaccines and escape from pre-existing anti-adenovirus antibodies. Mol Ther (2008) 16:154–62. 10.1038/sj.mt.6300306 17848961

[33] Miller BC Sen DR Al Abosy R Bi K Virkud YV LaFleur MW Subsets of exhausted CD8 + T cells differentially mediate tumor control and respond to checkpoint blockade. Nat Immunol (2019) 20:326–36. 10.1038/s41590-019-0312-6 PMC6673650 30778252

[34] McNamara HA Cai Y Wagle MV Sontani Y Roots CM Miosge LA Up-regulation of LFA-1 allows liver-resident memory T cells to patrol and remain in the hepatic sinusoids. Sci Immunol (2017) 2(9):1–10. 10.1126/sciimmunol.aaj1996 PMC5505664 28707003

[35] Fernandez-Ruiz D Ng WY Holz LE Ma JZ Zaid A Wong YC Liver-Resident Memory CD8+ T Cells Form a Front-Line Defense against Malaria Liver-Stage Infection. Immunity (2016) 45:889–902. 10.1016/j.immuni.2016.08.011 27692609

[36] Gerlach C Moseman EA Loughhead SM Alvarez D Zwijnenburg AJ Waanders L The Chemokine Receptor CX3CR1 Defines Three Antigen-Experienced CD8 T Cell Subsets with Distinct Roles in Immune Surveillance and Homeostasis. Immunity (2016) 45:1270–84. 10.1016/j.immuni.2016.10.018 PMC5177508 27939671

[37] Böttcher JP Beyer M Meissner F Abdullah Z Sander J Höchst B Functional classification of memory CD8 + T cells by CX 3 CR1 expression. Nat Commun (2015) 6(1):8306. 10.1038/ncomms9306 26404698 PMC4667439

[38] Li D Molldrem JJ Ma Q LFA-1 regulates CD8+ T cell activation via T cell receptor-mediated and LFA-1-mediated Erk1/2 signal pathways. J Biol Chem (2009) 284:21001–10. 10.1074/jbc.M109.002865 PMC2742865 19483086

[39] Wang Y Li D Nurieva R Yang J Sen M Carreño R LFA-1 affinity regulation is necessary for the activation and proliferation of naive T cells. J Biol Chem (2009) 284:12645–53. 10.1074/jbc.M807207200 PMC2675993 19297325

[40] Rissiek B Lukowiak M Raczkowski F Magnus T Mittrücker H-W Koch-Nolte F In Vivo Blockade of Murine ARTC2.2 During Cell Preparation Preserves the Vitality and Function of Liver Tissue-Resident Memory T Cells. Front Immunol (2018) 9:1580. 10.3389/fimmu.2018.01580 30038627 PMC6046629

[41] Rissiek B Haag F Boyer O Koch-Nolte F Adriouch S P2X7 on mouse T cells: One channel, many functions. Front Immunol (2015) 6:204. 10.3389/fimmu.2015.00204 26042119 PMC4436801

[42] Borges Da Silva H Beura LK Wang H Hanse EA Gore R Scott MC The purinergic receptor P2RX7 directs metabolic fitness of long-lived memory CD8+ T cells. Nature (2018) 559:264–8. 10.1038/s41586-018-0282-0 PMC6054485 29973721

[43] Bantug GR Fischer MG Grählert J Balmer ML Develioglu L Steiner R ER contact sites are immunometabolic hubs that orchestrate the rapid recall response of memory CD8 + T cells. Immunity (2018) 48:542–55. 10.1016/j.immuni.2018.02.012.Mitochondria PMC6049611 29523440

[44] Desdín-Micó G Soto-Heredero G Mittelbrunn M Mitochondrial activity in T cells. Mitochondrion (2018) 41:51–7. 10.1016/j.mito.2017.10.006 29032101

[45] Dan Dunn J Alvarez LAJ Zhang X Soldati T Reactive oxygen species and mitochondria: A nexus of cellular homeostasis. Redox Biol (2015) 6:472–85. 10.1016/j.redox.2015.09.005 PMC4596921 26432659

[46] Klein Geltink RI O’Sullivan D Corrado M Bremser A Buck MD Buescher JM Mitochondrial Priming by CD28. Cell (2017) 171:385–97.e11. 10.1016/j.cell.2017.08.018 28919076 PMC5637396

[47] Buck MD O’Sullivan D Pearce EL O’Sullivan D Pearce EL T cell metabolism drives immunity. J Exp Med (2015) 212:1345–60. 10.1084/jem.20151159 PMC4548052 26261266

[48] Frauwirth KA Riley JL Harris MH Parry RV Rathmell JC Plas DR The CD28 Signaling Pathway Regulates Glucose Metabolism. Immunity (2002) 16:769–77. 10.1016/S1074-7613(02)00323-0 12121659

[49] Esensten JH Helou YA Chopra G Weiss A Bluestone JA CD28 Costimulation: From Mechanism to Therapy. Immunity (2016) 44:973–88. 10.1016/j.immuni.2016.04.020 PMC4932896 27192564

[50] Masopust D Soerens AG Tissue-Resident T Cells and Other Resident Leukocytes. Annu Rev Immunol (2019) 37:521–46. 10.1146/annurev-immunol-042617-053214 PMC7175802 30726153

[51] Steinbach K Vincenti I Merkler D Resident-Memory T Cells in tissue-restricted immune responses: For better or worse? Front Immunol (2018) 9:2827. 10.3389/fimmu.2018.02827 30555489 PMC6284001

[52] Utzschneider DTT Charmoy M Chennupati V Pousse L Ferreira DPP Calderon-Copete S T Cell Factor 1-Expressing Memory-like CD8+ T Cells Sustain the Immune Response to Chronic Viral Infections. Immunity (2016) 45:415–27. 10.1016/j.immuni.2016.07.021 27533016

[53] Danilo M Chennupati V Silva JG Siegert S Held W Suppression of Tcf1 by Inflammatory Cytokines Facilitates Effector CD8 T Cell Differentiation. Cell Rep (2018) 22:2107–17. 10.1016/j.celrep.2018.01.072 29466737

[54] Li Y Tang L Guo L Chen C Gu S Zhou Y CXCL13-mediated recruitment of intrahepatic CXCR5+CD8+ T cells favors viral control in chronic HBV infection. J Hepatol (2020) 72:420–30. 10.1016/j.jhep.2019.09.031 31610223

[55] Jin Y Lang C Tang J Geng J Song HK Sun Z CXCR5+ CD8+ T cells could induce the death of tumor cells in HBV-related hepatocellular carcinoma. Int Immunopharmacol (2017) 53:42–8. 10.1016/j.intimp.2017.10.009 29032029

[56] Jiang H Li L Han J Sun Z Rong Y Jin Y CXCR5+ CD8+ T Cells Indirectly Offer B Cell Help and Are Inversely Correlated with Viral Load in Chronic Hepatitis B Infection. DNA Cell Biol (2017) 36:321–7. 10.1089/dna.2016.3571 28157399

[57] Leong YA Chen Y Ong HS Wu D Man K Deleage C CXCR5 + follicular cytotoxic T cells control viral infection in B cell follicles. Nat Immunol (2016) 17(10):1187–96. 10.1038/ni.3543 27487330

[58] Mackay LK Minnich M Kragten NAMM Liao Y Nota B Seillet C Hobit and Blimp1 instruct a universal transcriptional program of tissue residency in lymphocytes. Science (80-) (2016) 352:459–63. 10.1126/science.aad2035 27102484

[59] MacKay LK Rahimpour A Ma JZ Collins N Stock AT Hafon ML The developmental pathway for CD103+ CD8+ tissue-resident memory T cells of skin. Nat Immunol (2013) 14:1294–301. 10.1038/ni.2744 24162776

[60] Nicolas W Cortes-Penfield Barbara W Trautner RJ Endogenous Nur77 is a specific indicator of antigen receptor signaling in human T and B cells. Physiol Behav (2017) 176:139–48. 10.1016/j.physbeh.2017.03.040

[61] Chen L Flies DB Molecular mechanisms of T cell co-stimulation and co-inhibition. Nat Rev Immunol (2013) 13:227–42. 10.1038/nri3405 PMC3786574 23470321

[62] Kamphorst AO Wieland A Nasti T Yang S Zhang R Barber DL Rescue of exhausted CD8 T cells by PD-1 – targeted therapies is CD28-dependent. Science (2017) 355:1423–7. 10.1016/J.IMMUNI.2018.02.012 PMC5595217 28280249

[63] Chen Z Ji Z Ngiow SF Manne S Cai Z Huang AC TCF-1-Centered Transcriptional Network Drives an Effector versus Exhausted CD8 T Cell-Fate Decision. Immunity (2019) 51(5):840–55. 10.1016/J.IMMUNI.2019.09.013 PMC6943829 31606264

[64] Penaloza-MacMaster P Rasheed AU Iyer SS Yagita H Blazar BR Ahmed R Opposing Effects of CD70 Costimulation during Acute and Chronic Lymphocytic Choriomeningitis Virus Infection of Mice. J Virol (2011) 85:6168–74. 10.1128/jvi.02205-10 PMC3126534 21507976

[65] Wieland D Kemming J Schuch A Emmerich F Knolle P Neumann-Haefelin C TCF1+ hepatitis C virus-specific CD8+ T cells are maintained after cessation of chronic antigen stimulation. Nat Commun (2017) 8:1–13. 10.1038/ncomms15050 28466857 PMC5418623

[66] Klein Geltink RI Kyle RL Pearce EL Unraveling the Complex Interplay Between T Cell Metabolism and Function. Annu Rev Immunol (2018) 36:461–88. 10.1146/annurev-immunol-042617-053019 PMC6323527 29677474

[67] Mills EL Kelly B O’Neill LAJ Mitochondria are the powerhouses of immunity. Nat Immunol (2017) 18:488–98. 10.1038/ni.3704 28418387

[68] Buck MDD O’Sullivan D Klein Geltink RII Curtis JDD Chang CH Sanin DEE Mitochondrial Dynamics Controls T Cell Fate through Metabolic Programming. Cell (2016) 166:63–76. 10.1016/j.cell.2016.05.035 27293185 PMC4974356

[69] Lang PA Xu HC Grusdat M McIlwain DR Pandyra AA Harris IS Reactive oxygen species delay control of lymphocytic choriomeningitis virus. Cell Death Differ (2013) 20:649–58. 10.1038/cdd.2012.167 PMC3595491 23328631

[70] Schieber M Chandel NS ROS function in redox signaling and oxidative stress. Curr Biol (2014) 24:R453–62. 10.1016/j.cub.2014.03.034 PMC4055301 24845678

