# Supplementary material for: Improved Functionality of Exhausted Intrahepatic CXCR5+ CD8+ T Cells Contributes to Chronic Antigen Clearance Upon Immunomodulation
Source: Front Immunol. 2021 Feb 3;11:592328. doi: 10.3389/fimmu.2020.592328 (PMC7886981; doi:10.3389/fimmu.2020.592328)
Supplement: Supplementary file 1 [file Presentation_1.pdf]

Supplementary Data

Improved functionality of exhausted intrahepatic CXCR5+ CD8+ T cells contributes to chronic antigen clearance upon immunomodulation

Kingsley Gideon Kumashie, Marcin Cebula, Claudia Hagedorn, Florian Kreppel, Marina C. Pils, Friedrich Koch-Nolte, Björn Rissiek and Dagmar Wirth

Supplementary Fig. 1: High Ova antigen induces dysfunctional T cell response in the liver

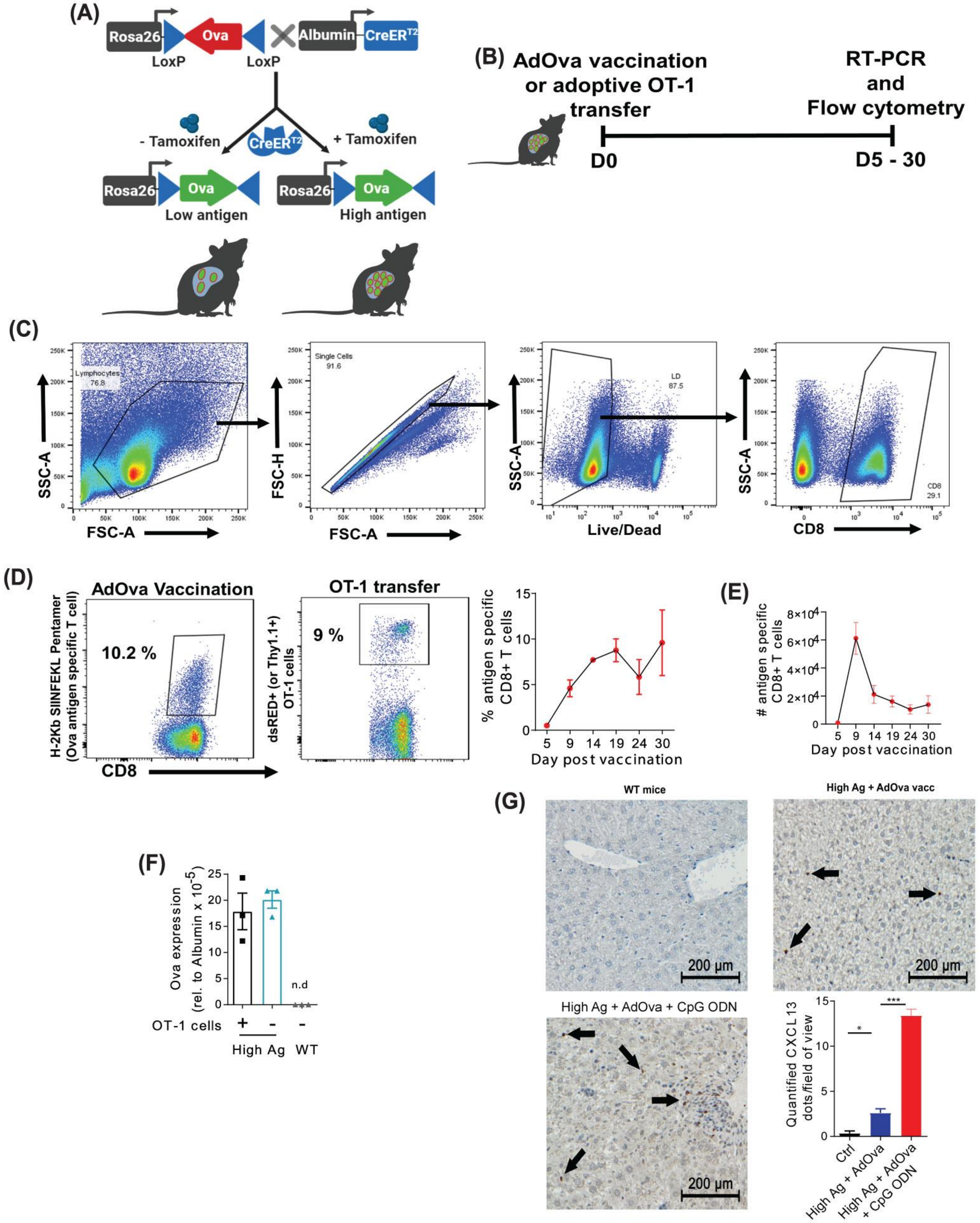

(**a**) Diagrammatic representation of the OvaXCre mouse model. The mouse model comprise of an Ova antigen gene which is inserted in an antisense direction to the endogenous Rosa26 promoter

and flanked by inversely oriented loxP sites (21). The Tam dependent CreERT2 recombinase is under the control of the hepatocyte specific albumin promotor. Without Tam administration, ca. 10 % of hepatocytes express Ova referred to as low antigen whereas upon induction with 50µg Tam, the frequency increases to ca. 50 % called high antigen (22). (**b**) Schematic representation of the experimental set-up. High antigen mice were either vaccinated with AdOva or adoptively infused with 3 – 5 x 10^6 OT-1 cells. Liver non-parenchyma cells were isolated from high antigen mice at various time points, phenotyped for antigen specific CD8+ T cells and analyzed by flow-cytometry. qRT-PCR was performed to assess antigen load D21 post OT-1 transfer. (**c**) Representative gating strategy used to identify CD8+ T cells from which Ova-antigen specific T cell gates were defined in (d). Lymphocyte gate was defined followed by single cell gating, live cells and CD8+ T cell gate. (**d**) Representative dot plot gates of Ova pentamer positive T cells in the liver D21 post AdOva vaccination (left) and dsRED+ or Thy1.1+ antigen specific T cells upon adoptive OT-1 cell transfer (middle), and the kinetic of antigen specific T cells (right) over 30 days period after AdOva vaccination. (**e**) Absolute number of antigen specific T cells in the liver from (d). (**f**) Relative Ova expression in the liver of high antigen mice transferred with OT-1 cells (black bar) and without OT-1 cells (light blue bar). RNA was isolated from liver slices of the respective treated condition D21 post OT-1 transfer and subjected to quantitative RT-PCR. The quantified Ova expression was normalized to the hepatocyte specific (albumin) housekeeping gene. WT mice were used as negative control. (**g**) Representative immune histological staining of CXCL13 in the liver of non-AdOva vaccinated WT mice and vaccinated high Ova antigen mice as wells as vaccinated high antigen mice *i.v* injected with CpG ODN on D3 post vaccination. Liver samples were harvested on D9 post vaccination. 3 µm thick sections of formalin-fixed, paraffin-embedded samples were produced according to standard laboratory procedures. Sections were de-paraffinized, treated with heat-mediated antigen retrieval, stained with polyclonal rabbit-anti-mouse CXCL-13 (Bioss, bs-4509R) and counterstained with hematoxylin. The quantification was based on three mice per condition with 10 fields of view per mice.

Supplementary Fig. 2: Tissue resident capacity of exhausted Ova-specific CD8+ T cell subset in the liver

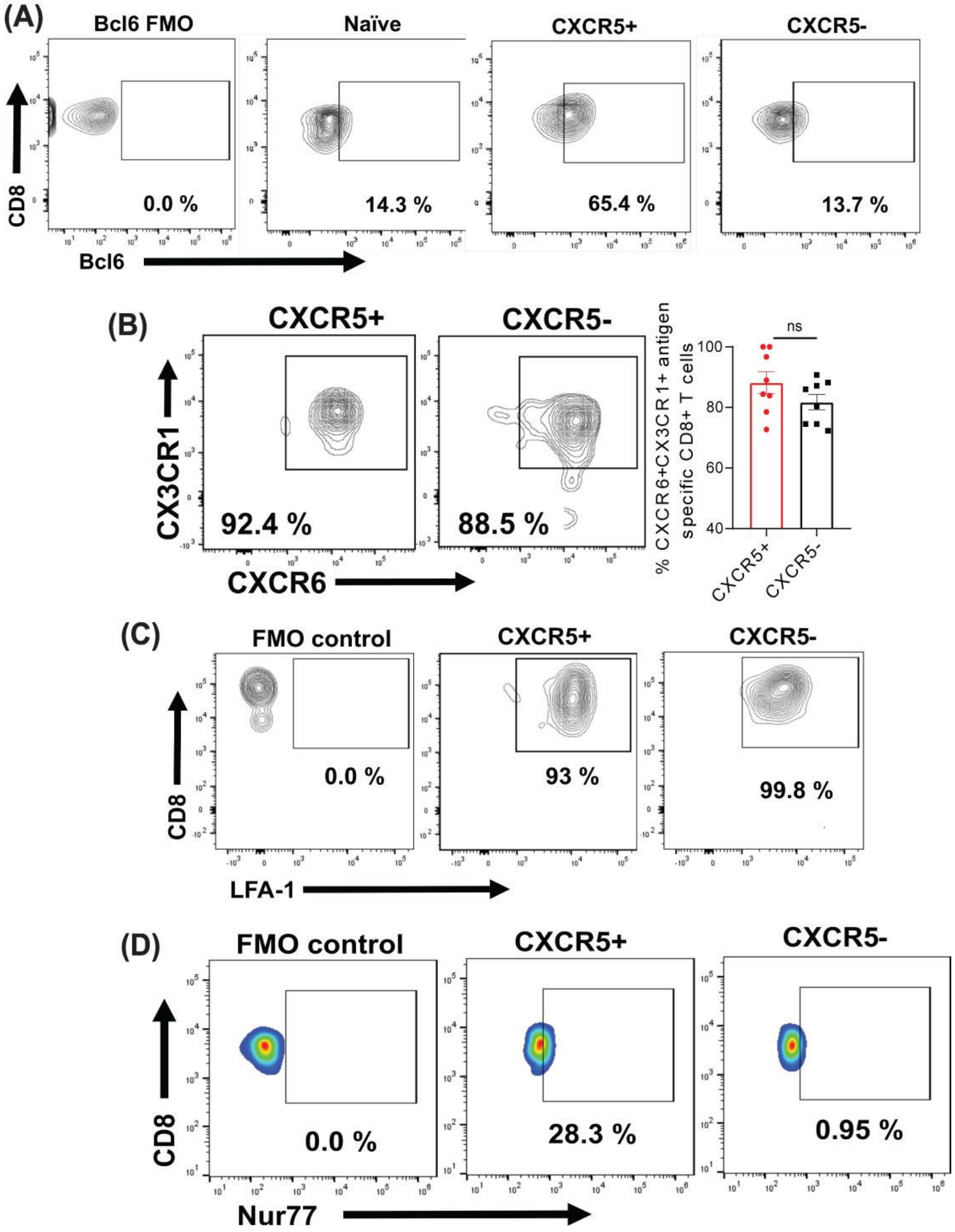

(**a, c**) Representative dot plots of (**a**) Bcl6 and (**c**) LFA-1 expressing cells. (**b**) Representative dot plot (left) and summary (right) of CXCR6 and CX3CR1 on Ova-specific CXCR5+ and CXCR5- T cells in the liver. (**d**) Representative dot plots of Nur77 expressing cells.

Supplementary Fig 3: Ova-specific CXCR5+ T cells express higher frequency of memory and activation markers

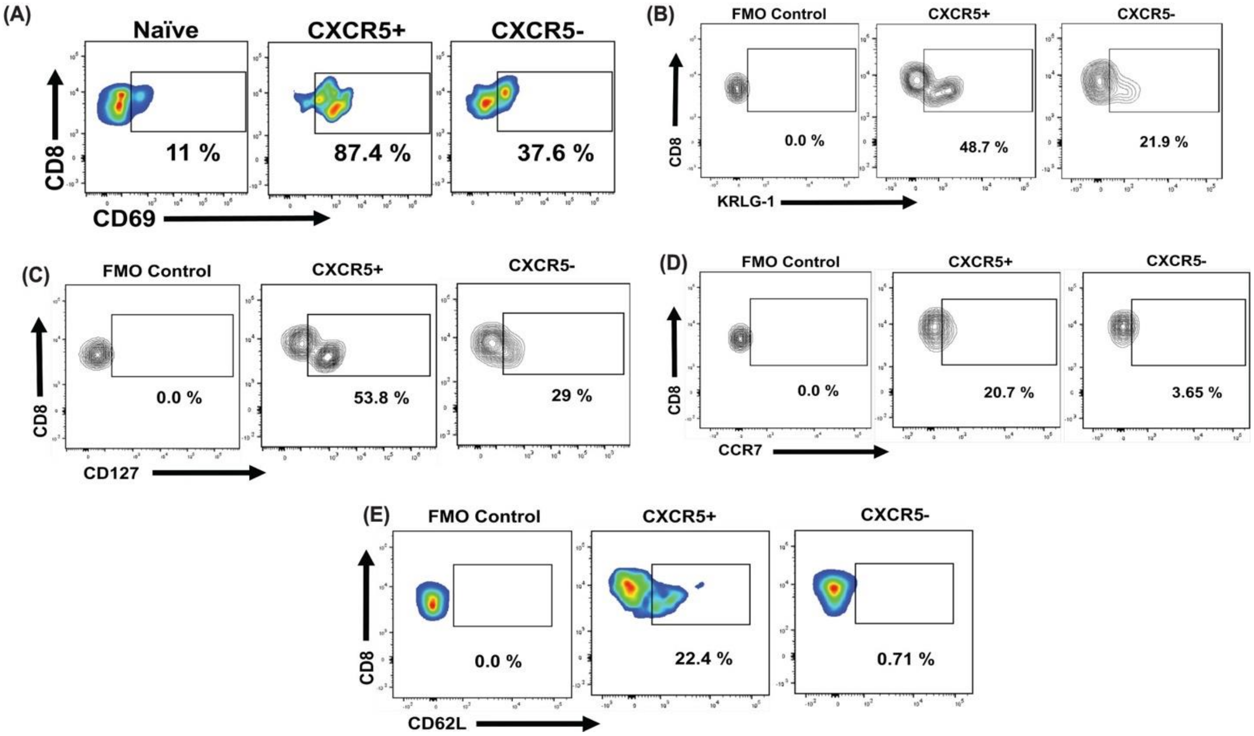

Additional data, referring to Figure 2a-i.

(**a - e**) Representative dot plot of (**a**) CD69, (**b**) KLRG-1, (**c**) CD127, (**d**) CCR7 and (**e**) CD62L expression.

Supplementary Fig 4: Expression frequencies and intensities of exhaustion markers

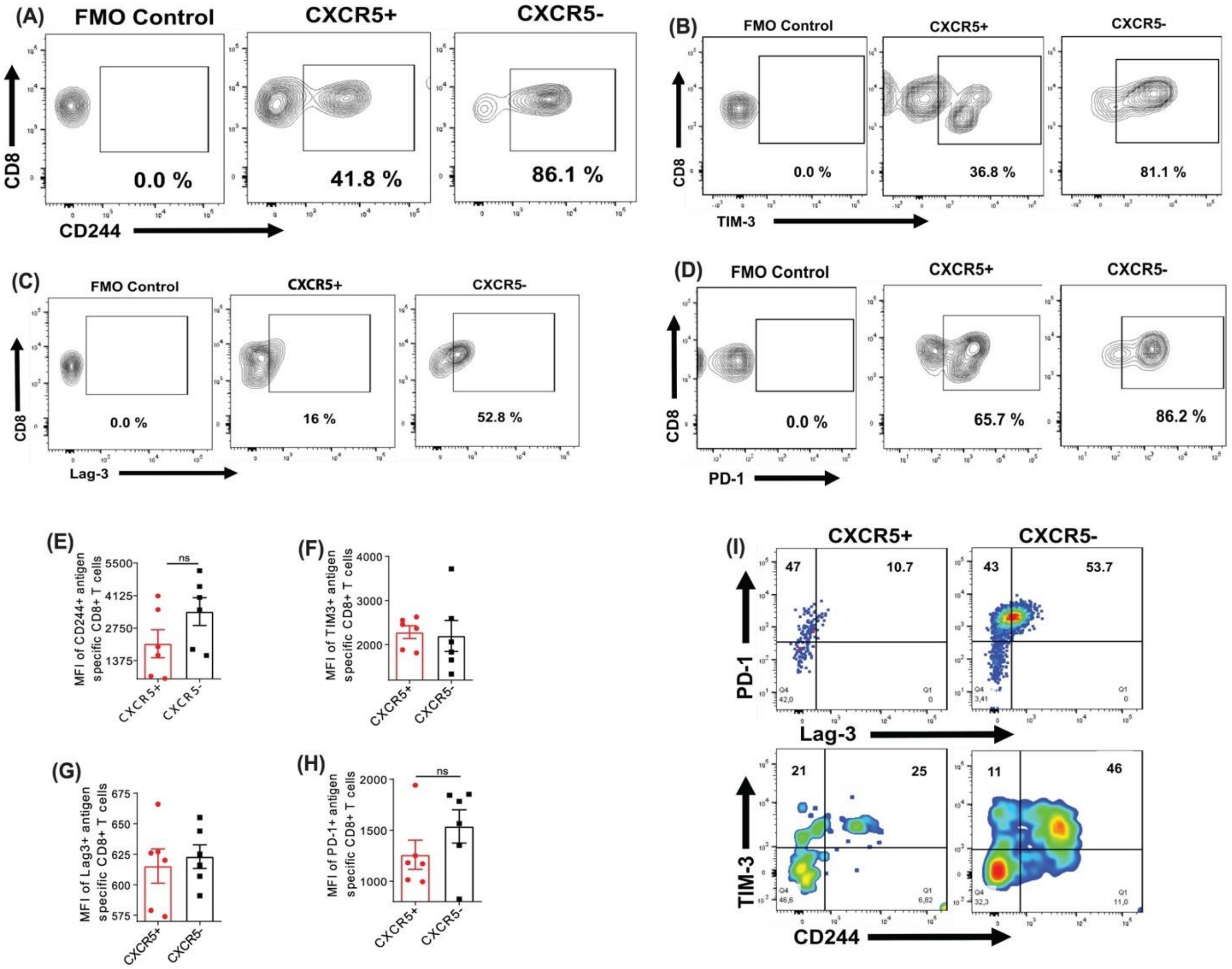

Additional data, referring to Figure 2j-m.

(**a – d**) Representative dot plots of (**a**) CD244, (**b**) TIM-3, (**c**) Lag-3 and (**d**) PD-1 expression. (**e – h**) MFI of (**e**) CD244, (**f**) TIM-3, (**g**) Lag-3 and (**h**) PD-1 expression on Ova-specific CXCR5+ and CXCR5- T cells. **(i)** Representative dot plot of PD-1+Lag-3+ and TIM-3+CD244+ expression on CXCR5+ and CXCR5- T cells. The numbers within the quadrants refer to the frequencies of cells.

Supplementary Fig. 5: Expression of effector molecules

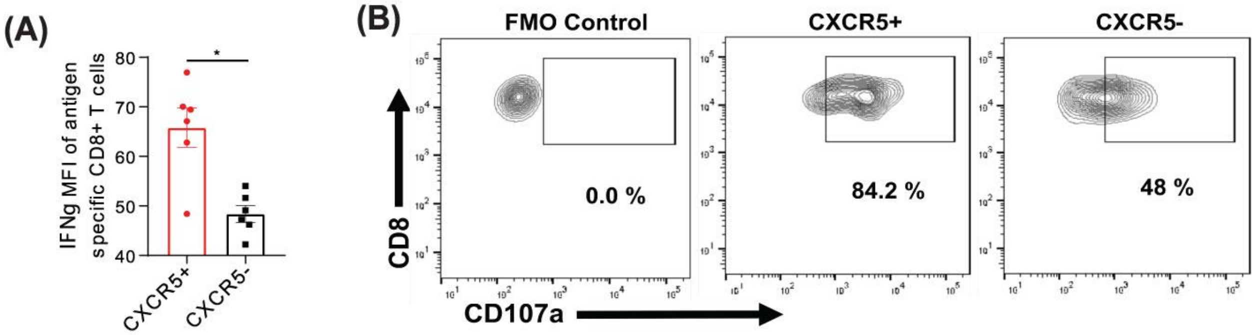

Additional data, referring to Figure 3.
(a) Representative dot plot of CD107a expression.

Supplementary Fig 6: Representative dot plots of P2X7R, TMRE, Glut-1, Ki67 and CD28 expression

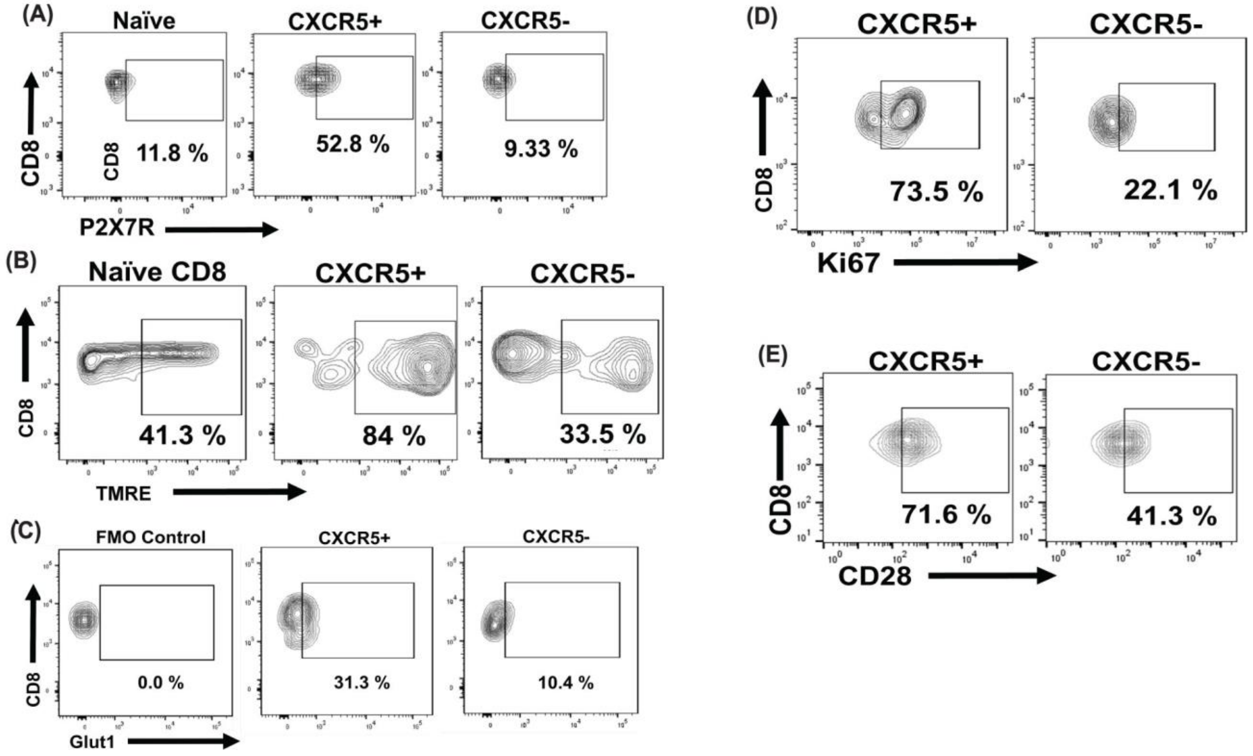

Additional data, referring to Figure 4.
Representative dot plots of (a) P2X7R expression (b) TMRE accumulation (c) Glut1 expression (d) Ki67 expression, with a gate marking Ki67^{hi} cells and (e) CD28 expression are indicated

Supplementary Table: List of antibodies

Extracellular markers

Antigen	Fluorophore	Dilution factor	Clone	Distributor
CD8	Percp-Cy5.5	400	53-6.7	Biolegend
CD8	AF647	1600	53-6.7	Biolegend
CD127	FITC	200	A7R34	Biolegend
CCR7	PE/Cy7	50	4B12	Biolegend
TIM-3	BV785	300	RMT3-23	Biolegend
PD-1	FITC	300	J43	eBiosciences
PD-1	PE	300	J43	eBiosciences
Lag-3	APC	300	eBioC9B7W	eBiosciences
CD62L	BV785	300	MEL-14	Biolegend
CXCR5	BV421	100	L138D7	Biolegend
KLRG-1	BV711	400	2F1/KLRG1	Biolegend
CD244 (2B4)	AF647	200	M2B4(B6)458.1	Biolegend
CD44	APC	500	IM7	Biolegend
H-2kb SIINFEKL	PE	100	F093-2A-E	ProImmune
CD69	FITC	300	H1.2F3	eBiosciences
CD11a (LFA-1)	AF647	300	M17/4	Biolegend
P2X7R	PE/Cy7	300	1F11	Biolegend
CD28	PE/Cy7	150	37.51	Biolegend
Thy1.1	PE	200	HIS51	eBiosciences
CD19	APC/Cy7	200	ID3	BD Biosciences
CD28	PE/Cy7	200	37.51	Biolegend
Thy1.1	APC	200	HIS51	eBiosciences

Transcription factors

Antigen	Fluorophore	Dilution factor	Clone	Distributor
Bcl6	AF647	100	IG191E/A8	Biolegend
Nur77	AF488	50	12.14	eBiosciences
Ki67	AF647	50	M2B4(B6)458.1	Biolegend
TCF-1/TCF-7	PE	50	S33-966	BD Biosciences

Intracellular markers

Antigen	Fluorophore	Dilution factor	Clone	Distributor
Granzyme b(GZMB)	PE/Cy7	200	NGZB	eBiosciences
IFNγ	FITC	300	554411	Biolegend
TNFα	APC	300	MP6-XT22	Biolegend
CD107a	FITC	150	1D4B	Biolegend
Glut-1	AF647	300	Ab195020	Abcam
